# Global distribution of single amino acid polymorphisms in *Plasmodium vivax* Duffy-binding-like domain and implications for vaccine development efforts

**DOI:** 10.1098/rsob.200180

**Published:** 2020-09-30

**Authors:** Payal Mittal, Siddhartha Mishra, Sonalika Kar, Veena Pande, Abhinav Sinha, Amit Sharma

**Affiliations:** 1 Molecular Medicine Group, International Centre for Genetic Engineering and Biotechnology, New Delhi, 110067, India; 2 ICMR-National Institute of Malaria Research, New Delhi, 110077, India; 3 Department of Biotechnology, Kumaun University, Nainital, Uttarakhand, 263001 India

**Keywords:** *Plasmodium vivax*, erythrocyte-binding proteins, Duffy-binding-like domain, proteomic polymorphism, antibody–antigen interaction, vaccine development

## Abstract

*Plasmodium vivax* (*Pv*) malaria continues to be geographically widespread with approximately 15 million worldwide cases annually. Along with other proteins, Duffy-binding proteins (DBPs) are used by plasmodium for RBC invasion and the parasite-encoded receptor binding regions lie in their Duffy-binding-like (DBL) domains—thus making it a prime vaccine candidate. This study explores the sequence diversity in *Pv*DBL globally, with an emphasis on India as it remains a major contributor to the global *Pv* malaria burden. Based on 1358 *Pv*DBL protein sequences available in NCBI, we identified 140 polymorphic sites within 315 residues of *Pv*DBL. Alarmingly, country-wise mapping of SAAPs from field isolates revealed varied and distinct polymorphic profiles for different nations. We report here 31 polymorphic residue positions in the global SAAP profile, most of which map to the *Pv*DBL subdomain 2 (*α*1–*α*6). A distinct clustering of SAAPs distal to the DARC-binding sites is indicative of immune evasive strategies by the parasite. Analyses of *Pv*DBL-neutralizing antibody complexes revealed that between 24% and 54% of interface residues are polymorphic. This work provides a framework to recce and expand the polymorphic space coverage in *Pv*DBLs as this has direct implications for vaccine development studies. It also emphasizes the significance of surveying global SAAP distributions before or alongside the identification of vaccine candidates.

## 1. Background

Malaria remains a serious public health concern for large swathes of the world. Of the five plasmodia species that cause human malaria, *Plasmodium falciparum* (*Pf*) is responsible for most mortality, but in Asia and South America, *Plasmodium vivax* (*Pv*) is also a significant cause of morbidity [1]. *Pv* has the ability to form hypnozoites, a dormant liver stage parasite, making its elimination difficult [2]. Due to the unavailability of diagnostic tools for dormant *Pv* liver stages, relapse remains a major source of recurrent *Pv* malaria [2]. *Pv* preferably invades young reticulocytes and can quickly form gametocytes, and these facets complicate its control, leading to approximately 15 million annual cases worldwide [1–4]. Plasmodial invasion into RBCs triggers the symptomatic phase of its life cycle and is a key determinant of pathogenesis. The invasion of erythrocytes by *Plasmodium* merozoites is a complex, multistep process and requires specific receptor–ligand interactions [5–10]. Most of the parasite proteins involved in such interactions are potential targets for the human immune system and display extensive polymorphisms as a mechanism for immune evasion [11,12]. One such candidate for *Pv* is the cysteine-rich Duffy-binding protein (DBP), which is in a subset of the erythrocyte-binding antigens (EBAs) [13,14]. DBP helps the parasite bind to Duffy antigen receptor for chemokines (DARC) for invasion and entry—known as the DARC-dependent invasion pathway [8,15,16]. There are also DARC-independent invasion pathways as reported for in *Pf* and *Plasmodium knowlesi* (*Pk*) [9,17]. DARC is present on the surface of endothelial cells and erythrocytes [18]. It is a hepta-helical transmembrane protein and is involved in pro-inflammatory responses as it is a promiscuous receptor for chemokines/cytokines [18,19]. *Pv* hijacks human DARC for invasion and uses the parasite-encoded DBL for DARC recognition [20].

*P. vivax* Duffy-binding protein (*Pv*DBP) is composed of seven regions/domains, has a molecular weight of approximately 140 kDa and is a type-1 integral membrane protein [5]. Region II of *Pv*DBP (*Pv*DBP-II) is known as the Duffy-binding-like domain (*Pv*DBL) and this domain contains the critical DARC-binding motifs [9,21–25]. *Pv*DBL and its *Pk*DBL orthologues are boomerang-shaped, monomeric structures with three distinct subdomains [11,23]. *Pv*DBL contains twelve cysteine residues that form intra-domain disulfide bonds and these are largely conserved within DBLs [11,24,25]. Of the three subdomains in *Pv*DBL, subdomain 1 has been shown to be dispensable for DBL–DARC interaction [11,14]. The critical DARC-binding residues have been mapped between cysteines 4 and 7 [21,24]. Subdomain 2 contains two sites for DARC binding—Site1 and Site2—as described recently [25]. These two sites have been annotated based on comprehensive analyses of crystal structure information, mutagenesis studies and RBC-binding assays [11,18,22,23,25–32]. The two sites contain conserved residues that are surface exposed and they probably play central roles in DARC binding via recognition of the sulfated tyrosines [11,14,23,25]. Site1 residues include K297, K301, R304 and K378, while K273, R274 and Q356 constitute Site2 [25]. DARC binding is facilitated via atomic interactions of residues within Site1 and Site2 with post-translationally modified (sulfated) Tyr41 and Tyr30 on DARC peptide, respectively [25,28]. An interaction model of *Pv*DBP-II-DARC binding has been reported earlier [25]. Other non-polar and hydrophobic residues that are important for DBL–DARC interaction to be maintained include K289, Y295, N296, F299, Y363, K366, K367, L369, F373 and I376 [11,27,31,32]. These are collectively referred to as DARC-associated binding residues (DaBR), while the sulfated tyrosine recognition ones at Site1 and Site2 are known as DARC-binding critical residues (DbCR) (see electronic supplementary material, figure S1).

In regions where *Pv* is endemic, naturally acquired immune responses against *Pv*DBL are associated with reduced risk of parasitaemia and lower *Pv* invasion into host RBCs—thus making *Pv*DBL a domain of interest [33–35]. Despite this, one of the major impediments for successful vaccine design to enable global protection is the extensive polymorphic nature of *Pv*DBP—especially of *Pv*DBL, which potentially indicates strategies of evasion from host immune responses [6,7,13,36–42]. Some of the polymorphisms in *Pv*DBL cause antigenic drift and hence are responsible for strain-specific immune responses [43]. Complicating these scenarios further are additional facets of *Pv* biology—several cases of *Pv* infection have now been observed in Duffy-negative individuals, suggesting that the parasite might have evolved an alternative pathway that is independent of DARC–DBL interaction for invasion [44–52]. Studies from Malagasy, Colombia and Ethiopia have also suggested that *Pv*DBP gene copy variations in the field are evident and indicative of *Pv* evolution [53–55]. Therefore, the development of *Pv*DBL-based vaccine seems to be fraught with several challenges—and here we highlight further reasons for scepticism in this direction.

**Figure 1.**
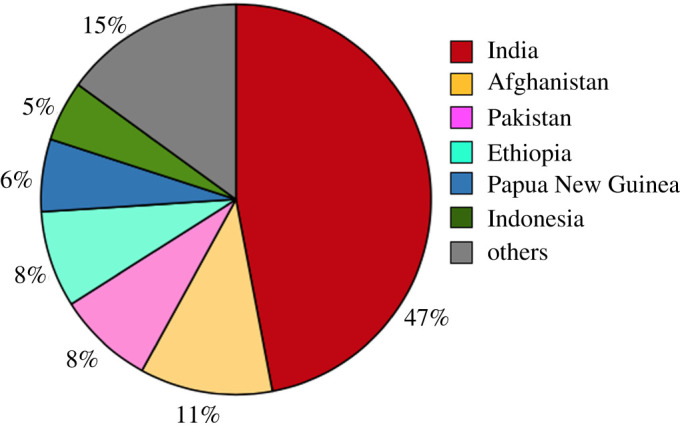
*Plasmodium vivax* malaria burden. Pie chart showing the distribution of *Pv* across the world. Source: Figure adapted from Figure 2.1 (*b*) in [56].

A thorough examination of *Pv*DBL sequences from global *Pv* isolates will provide a better understanding of how natural selection has shaped this antigen across different populations and continues to do so. India contributes approximately 47% of *Pv* malaria burden globally [56] (figure 1). We here provide both new *Pv*DBL sequences from across India and also use the *Pv*DBL sequences recorded in GenBank to assess the extent of non-synonymous single-nucleotide polymorphisms which give rise to single amino acid polymorphisms (SAAPs).

## 2. Methods

### 2.1. Sample collection

**Figure 2.**
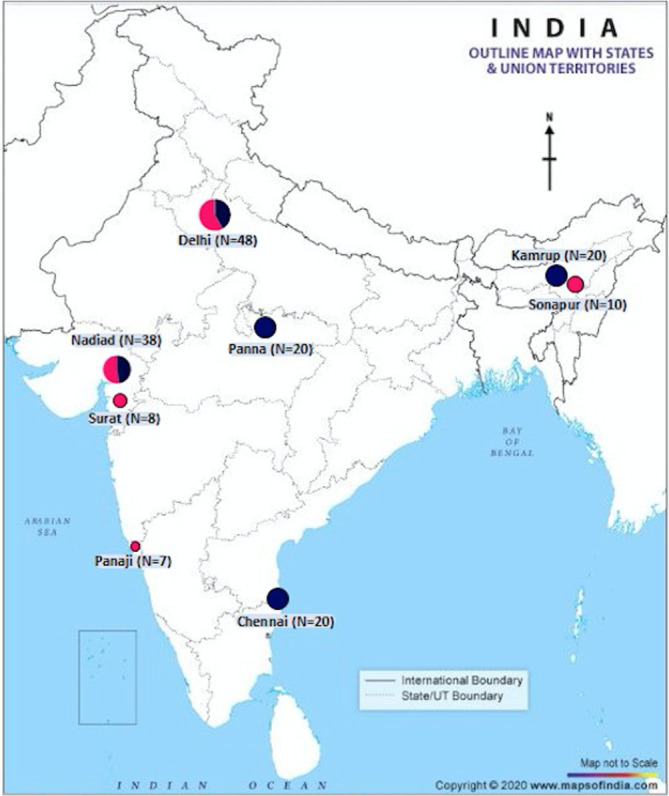
Sample collection within India. Sites are shown as coloured circles whose size is proportional to the number of samples collected. Dark blue circles show 98 samples collected between 2003 and 2006 from Kamrup (Assam, *n* = 20), Delhi (*n* = 20), Nadiad (Gujarat, *n* = 18), Panna (Madhya Pradesh, *n* = 20) and Chennai (Tamil Nadu, *n* = 20) [57]. Pink circles show 73 samples collected between 2014 and 2019 from Sonapur (Assam, *n* = 10), Delhi (*n* = 28), Panaji (Goa, *n* = 7), Surat and Nadiad (Gujarat, *n* = 8 and 20, respectively). Circles with dark blue and pink represent shared locations for both sources.

A total of 176 finger-pricked blood samples as dried blood spots were collected from adult patients showing symptoms of malaria between 2014 and 2019. The search for cases/samples included active (house-to-house visits) and passive (from malaria clinics, ICMR-NIMR field units and Malaria Parasite Bank of ICMR-NIMR) case detection from suspected individuals in four geographically distinct localities of India with variable *Pv* malaria epidemiology: Sonapur in Assam; Delhi; Panaji in Goa; and Nadiad and Surat in Gujarat (table 1). This study was approved by the Institutional Ethics Committee of ICMR-NIMR, New Delhi, India, and written informed consents were obtained before the samples were collected from all patients who participated in the study. The locations of the sample collection sites in India are shown in figure 2. In addition, all available *Pv*DBP-II DNA sequences (*n* = 100) corresponding to a part of the DBL domain (927 bp) collected from four states in India (figure 2 and table 1) were downloaded from NCBI (GenBank ID: FJ491142–FJ491241).

**Table 1.** Details of 171 *P. vivax* samples from India analysed in this study.

state	location & source	number of samples	GenBank ID
Assam	Kamrup^a^	20	FJ491203–22
	Sonapur^b^	10	MT502426–35
Delhi	New Delhi^a^	20	FJ491163–82
	New Delhi^b^	28	MT502436–63
Goa	Panaji^b^	7	MT502492–98
Gujarat	Nadiad^a^	18	FJ491183–5, FJ491188–202
	Nadiad^b^	20	MT502472–91
	Surat^b^	8	MT502464–71
Madhya Pradesh	Panna^a^	20	FJ491142, FJ491223–41
Tamil Nadu	Chennai^a^	20	FJ491143–62
**Total**		**171**	

^a^Sequences downloaded from NCBI sampled during 2003–2006.

^b^Sequences collected in this study during 2014–2019.

### 2.2. Amplification and sequencing of the Duffy-binding ligand domain of *Plasmodium vivax* Duffy-binding protein-II gene

For each collected blood sample (*n* = 176), genomic DNA was isolated using the QIAamp DNA mini kit (Qiagen, Germany) according to the manufacturer's instructions. Because both *Pf* and *Pv* occur in India in almost equal proportions [58], we confirmed the presence of *Pf* and *Pv* using nested polymerase chain reaction (PCR) assays with genus- and species-specific oligonucleotide primers based on the 18S rRNA gene [59]. Genomic DNA from only *Pv-*infected individuals (*n* = 73) was then used for further analysis. A 945 bp region hereafter referred to as *Pv*DBL, corresponding to nucleotide positions 992–1936 (corresponding amino acid positions 211–525) from the transcription start site in the *P. vivax* Salvador I reference sequence (GenBank ID: M37514), was amplified using 5′-3′ oligonucleotide pair *Pv*DBP-II-F (GCATGAGGGAAATTCTCGTA) and *Pv*DBP-II-R (GGAGATTCTACGCATGGAAA). The oligonucleotide primer was designed using the OligoAnalyzer Tool (IDT, IA, USA) and synthesized by Integrated DNA Technologies (IA, USA). PCR was carried out in a final volume of 25 µl, which included 0.4 µM of each oligonucleotide, 0.2 mM dNTP mix (GeNei, India) and 1U Taq DNA polymerase (GeNei, India) with 1X Taq Buffer A (GeNei, India) and 2 µl genomic DNA template. The PCR was performed in a Veriti 96-Well Thermal Cycler (ThermoFisher Scientific, USA) using the following conditions: initial denaturation at 95°C for 5 min, 35 cycles each of denaturation at 95°C for 1 min, annealing at 58°C for 1 min and extension at 72°C for 1 min 20 s, followed by a final extension at 72°C for 10 min. Approximately 5 µl of amplified DNA was fractionated using 1% w/v agarose gel (GeNei, India) in 1X TBE buffer at 75 volts for 45 min, along with 1 kb DNA marker (Promega, Madison, USA) to confirm the desired amplicon size (1146 bp). The gel was stained with 30 µg Ethidium Bromide (Promega, Madison, USA) and visualized on UVITEC gel documentation system (UVITEC, UK). Successfully amplified products were purified using 10 µl of amplified DNA, 10 U of Exonuclease-I (ThermoFisher Scientific, USA) and 1 U of Shrimp Alkaline Phosphatase (Applied Biosystems, ThermoFisher Scientific, USA) and 1 X Taq Buffer A (GeNei, India) in a thermal cycler at 37°C for 120 min, followed by enzyme inactivation at 85°C for 15 min. The purified DNA fragments (2 µl) and 0.8p M each of *Pv*DBP-II-F and *Pv*DBP-II-R were transferred to two different wells (for 2 X coverage) of MicroAmp Optical 96-Well Reaction Plate (ThermoFisher Scientific, USA). Finally, 0.5 µl of BigDye Terminator 3.1 Ready Reaction Mix and 2 µl 5 X sequencing buffer from BigDye Terminator v3.1 Cycle Sequencing Kit (ThermoFisher Scientific, USA) and ddH_2_O (adjustable to final volume of 10 µl) were added to the DNA-primer mix. The 96-well plate was then transferred to an ABI 3730XL DNA Analyser (Applied Biosystems, CA, USA) for Sanger sequencing at ICMR-National Institute of Malaria Research (ICMR-NIMR), New Delhi, India. The raw *Pv*DBL sequences were edited using EditSeq and SeqMan modules of the LASERGENE v7 computer program (DNASTAR, Madison, USA). The edited *Pv*DBL DNA sequences were deposited in GenBank under accession numbers MT502426–MT502498. Amino acid multiple sequence alignment of 171 sequences (73 sequenced *de novo* and 98 out of 100 downloaded) was performed with the ClustalW program in MEGA X and polymorphic residues were identified [60].

### 2.3. Global *Plasmodium vivax* Duffy-binding-like domain sequence retrieval, curation and SAAP analyses

The protein sequences under the labels of ‘*Plasmodium vivax* Duffy-binding-like domain’ and ‘*Plasmodium vivax* Duffy-Binding Protein Region II’ were retrieved from the NCBI GenBank database. All hypothetical, partial and unverified data entries were removed. This formed a final list of 1285 protein sequences to which 73 newly submitted sequences from India were added such that the entire set of 1358 protein sequences constitute the global dataset of *Pv*DBL sequences (table 2). All the sequences were classified on the basis of their respective country of collection. We divided our dataset into six sub-groups so as to represent different continental regions: Southeast Asia (India, Thailand, Republic of Korea, Malaysia, Myanmar and Sri Lanka), Oceania (Papua New Guinea), Americas (Brazil, Colombia and Mexico), Africa (Sudan and Uganda), Middle East (Iran) and Central Asia (Kyrgyz Republic). Protein sequences of all isolates from each region were aligned using Clustal Omega and scanned for polymorphic residues. The country/region-wise divided data points were simultaneously analysed to assess if any country/region-specific SAAPs were prominent. These data were then collated to allow for a global view of polymorphisms and to calculate the frequency of each SAAP. The SAAPs were classified as conservative or non-conservative on the basis of their physico-chemical properties. Analyses beyond this stage included residues that exhibited polymorphic nature with a minimum frequency of 0.5% in the global dataset of 1358 sequences. Those SAAPs with a minimum frequency of 0.5% but less than 5% in the global set are classified here as ‘minor’ whereas those with a frequency of 5% and above are classified as ‘major’ (median frequency of dataset = 5%). The total number of instances of SAAPs was also classified on the basis of occurrence within particular subdomains of *Pv*DBL to deduce the most variable subdomain of *Pv*DBL. We analysed the distribution of SAAPs on the three-dimensional structure of *Pv*DBL in relation to Site1 and Site2 [25]. The 4 mAb-bound *Pv*DBL structures in PDB (Protein Data Bank), purportedly with strain-transcending broadly neutralizing antibodies, were analysed using PDBePISA/PDBsum to identify the interfacial residues [63,68]. These data were finally contextualized to analyse residues that were polymorphic in the global SAAP dataset.

**Table 2.** Country-wise list of NCBI accession IDs of worldwide *Pv*DBL sequences.

country	nucleotide accession ID	protein accession ID	no. of sequences (*n*)	year of study	reference
Brazil	EU812839–960	ACJ01669–790	122	2003	[75]
	JQ405271–93, EU870443–445, KP036999–7006	AFV77927–949, ACJ64695–697, AKU77037–044	34	2003–2005	[37]
	MN223747–962	QIE13984–4199	216	2011–2018	[70]
Myanmar	JN255576–587	AFD18594–605	54	2004–2006	[39]
	MN233407–488 MN233489–573	QGQ33234–315 QGQ33316–400	82 85	2016–2017	[67]
Papua New Guinea	AF469515–602	AAL79043–130	88	2000	[61]
	AY970837–925	AAY34048–136	89	2000	[62]
	AF289635–653, AF289480–483, AF291096	AAG53617–634, AAG30847–850, AAG31571	22	2000	[36]
India	FJ491142–241	ACN69874–971	98	2003–2006	[57]
	MT502426–498	not available yet	73	2014–2019	current study
Sri Lanka	GU143914–4013	ACY91984–2083	100	1998–2000	[38]
Republic of Korea	AF220657–668	AAF25483–494	12	1996–1997	[64]
	JN989472–484	AFO42559–571	70	2005–2010	[40]
	AF215737, AF215738	AAG43989, AAG43990	2	1998	[71]
Sudan	MG805616–657	AXR85467–508	42	2014–2016	[74]
Mexico	KP759780–KP759813	AKS26850–883	34	2006–2007	[72]
Uganda	KX009537–560	ARJ54093–116	24	2016	unpublished
	JX174522–528	AGH67766–772	7	2012	unpublished
Thailand	EF219451, EF368159–180, EF379127–135	ABQ10597, ABR13991–4012, ABR08404–411	30	2002–2003	[65]
Iran	EU860428–438	ACJ54187–197	11	2000–2007	[73]
	KF751807–810	AHB23286–289	4	2013	[77]
	KF791921–926	AHV83759–764	6	2008–2012	[41]
	KF318358, KF318359	AHF22121, AHF22122	2	2008–2012	[76]
Malaysia	MF624859–876	AXB88414–431	18	2017	unpublished
Colombia	U50575–591	AAC47175–191	17	1996	[69]
Kyrgyz Republic	MK014215–230	AYN76785–800	16	2006	[42]
Total			1358	1996–2019	

## 3. Results

### 3.1. *Plasmodium vivax* Duffy-binding-like domain SAAP profile within India

Out of the 176 samples from India (collected between 2014 and 2019), 104 were positive for *Plasmodium* spp. (73 for *Pv* and 31 for *Pf*) and no mixed-species *Plasmodium* (*Pv* *+* *Pf*) infection was found. DNA from the 73 *P. vivax* mono-infected isolates were used for further analysis. Out of 100 sequences downloaded from NCBI, a total of 98 sequences were included in this study (sequences with GenBank IDs FJ491186 and FJ491187 were excluded as they had stop codons in their coding regions). Hence, a total of 171 sequences formed the total sample from India for this study (figure 2).

**Figure 3.**
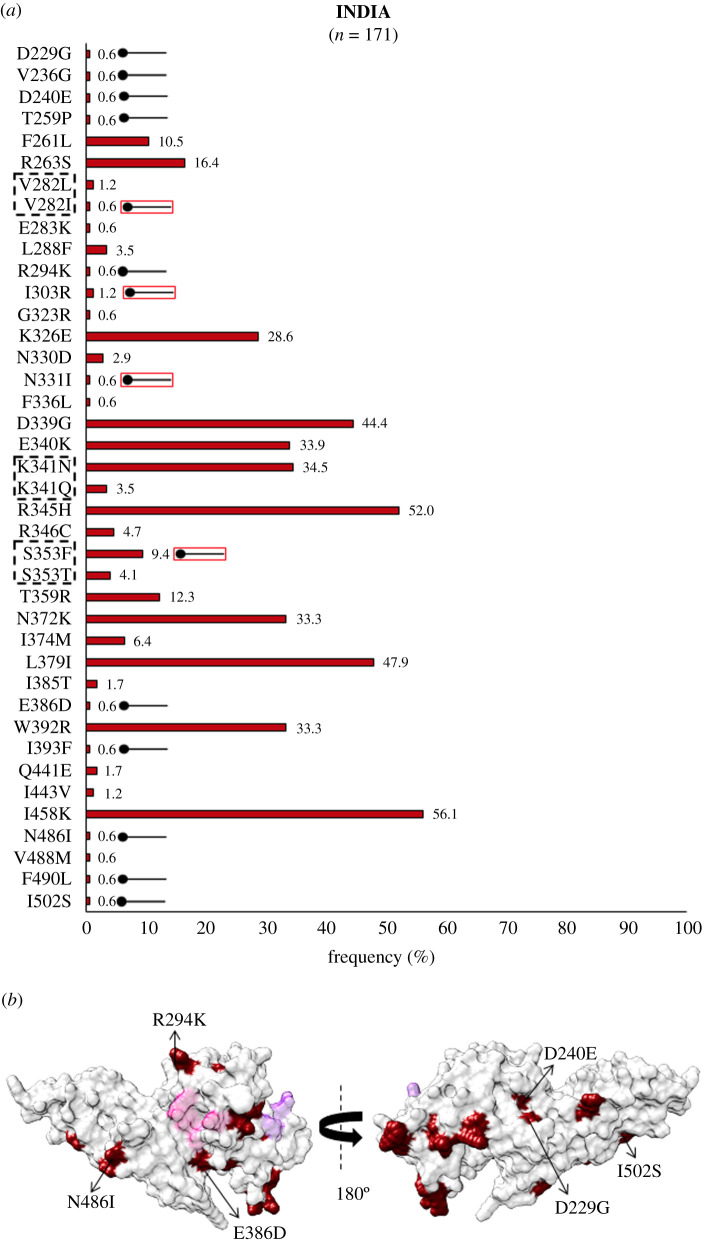
(*a*) *Pv*DBL SAAP Profile within India. Bar graph showing frequencies (dark red) of 40 SAAPs from 37 polymorphic residues of *Pv*DBL observed in 171 Indian isolates. Three trimorphic residue positions (282, 341 and 353) are outlined with a dashed rectangle. A total of 14 geographically exclusive SAAPs are observed in Indian isolates and are marked with a horizontal balloon symbol. Of these 14, 10 are exclusive while 4 are different SAAP forms of multivariant residues (V282I, I303R, N331I and S353F). These are marked with the help of a horizontal balloon outlined with red. (*b*) Structural position of Indian SAAPs in Site1 and Site2. Molecular surface representation of *Pv*DBL with all polymorphic residue positions found in Indian isolates (dark red) is shown in addition to Site1 (pink) and Site2 (purple) residues. Geographically exclusive polymorphic residues are labelled.

Protein sequence analysis of *Pv*DBL among 171 *Pv* isolates from India revealed that there were 37 polymorphic residue positions which contained 40 SAAPs, of which 24 SAAPs occurred with a frequency of 1% and above. Of these polymorphisms, amino acid changes at positions 282, 341 and 353 were trimorphic. SAAPs that occurred with a frequency less than 1% but were found to be exclusively present only in isolates from India include D229G, V282I, V236G, D240E, T259P, R294K, I303R, N331I, E386D, I393F, N486I, F490L and I502S. The SAAPs—V282I, I303R, N331I and S353F—were found to occur in multivariant forms globally but have one of those forms exclusive to isolates from India—as shown in figure 3*a*. Total of 75% SAAPs were non-conservative (30 of 40). E386D was found to occur within 5 Å radius of Site1, while the trimorphic mutation S353F/T occurs within 5 Å radius of Site2 (figure 3*b*). Considering that N372K, L379I, W392R and I458K either individually or in combination could affect *Pv*DBL antigenicity, the presence of such polymorphisms was sought [43]. I458K was found to occur with a high frequency (56%) as compared to N372K and W392R, which occurred with low frequencies (33% each). The N372K-L379I-W392R trio was found in 33% (56 of 171) of the total samples, while 22% (37 of 171) isolates bear the fourth polymorphic position I458K in addition to the trio. An insertion of a leucine between V429 and P430 in subdomain 3 was seen in 10.5% of isolates from India (18 of 171).

### 3.2. *Plasmodium vivax* Duffy-binding-like domain SAAPs profiles from other countries/regions

**Figure 4.**
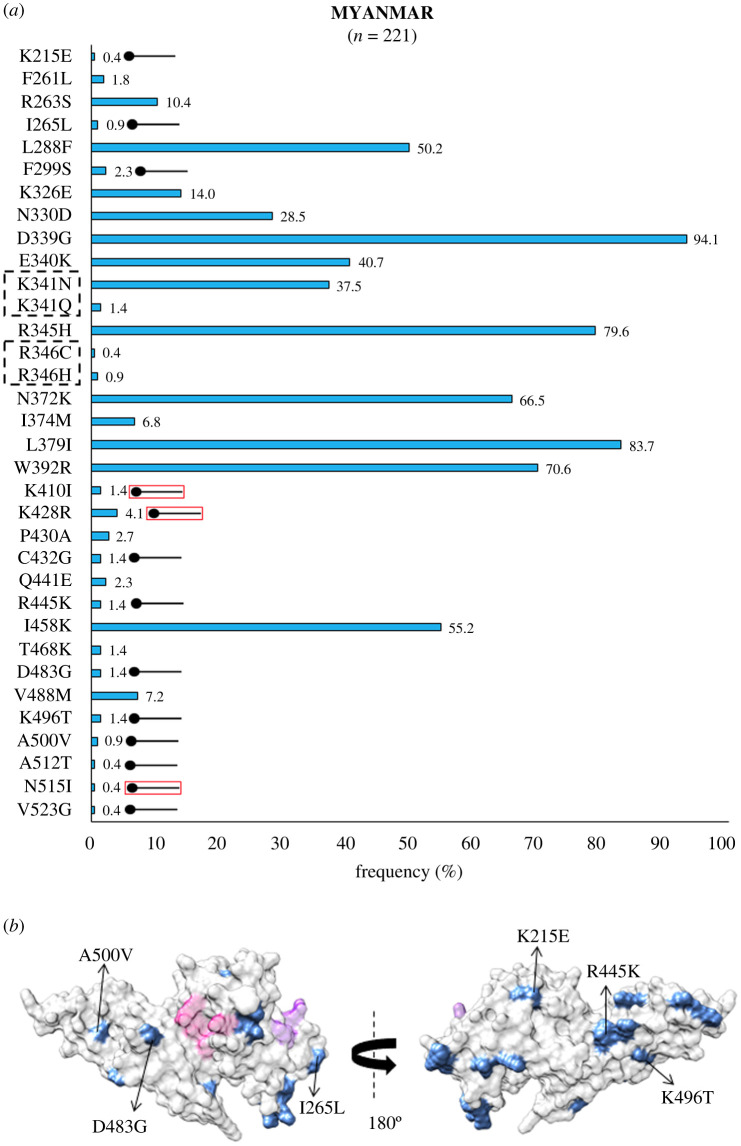
(*a*) SAAP profile within Myanmar. Bar graph showing frequencies (cornflour blue) of 34 SAAPs from 32 polymorphic residues of *Pv*DBL observed in 221 sequences from Myanmar. Two trimorphic residue positions (341 and 346) are outlined with a dashed rectangle. Thirteen geographically exclusive SAAPs observed in Myanmarian isolates are marked with a horizontal balloon symbol. Of those 13, 10 are exclusive polymorphic residues, while three are exclusive SAAP forms of multivariant residues (K410I, K428R and N515I) and are marked with the help of a horizontal balloon outlined with red. (*b*) Relative structural position of SAAPs within Myanmar with Site1 and Site2. Molecular surface representation of *Pv*DBL with all polymorphic residue positions found in Myanmarian isolates (cornflower blue) is shown in addition to Site1 (pink) and Site2 (purple) residues. Geographically exclusive polymorphic residues are labelled.

**Figure 5.**
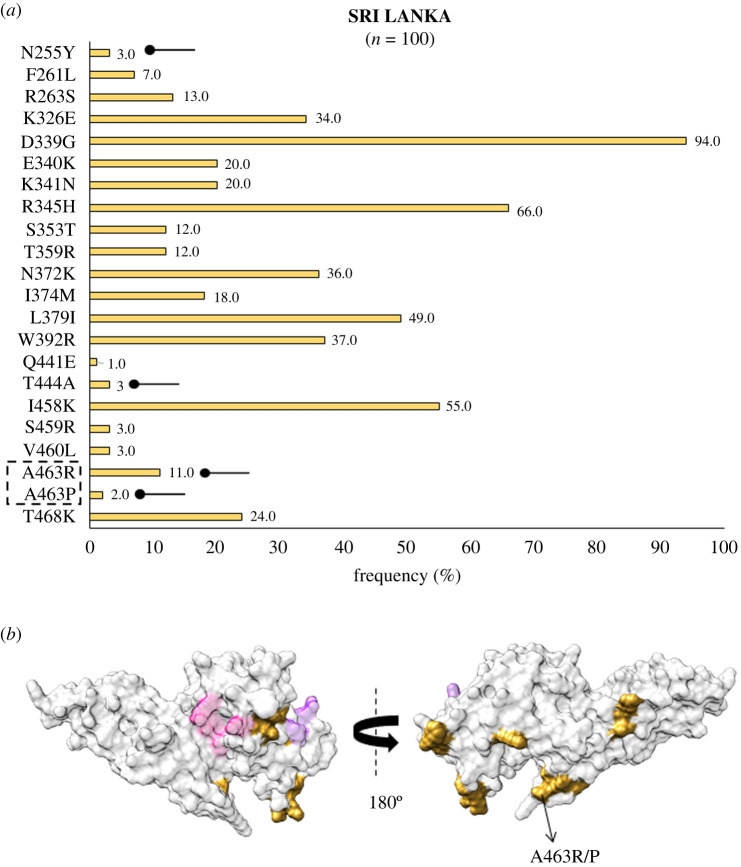
(*a*) Graph showing *Pv*DBL SAAP profile within Sri Lanka. Bar graph showing frequencies (golden rod) of 22 SAAPs from 21 polymorphic residues of *Pv*DBL observed in 100 sequences from Sri Lanka. One trimorphic residue at position 463 is outlined with a dashed rectangle. Four geographically exclusive SAAPs observed in Sri Lankan isolates are marked with a horizontal balloon symbol. (*b*) Relative structural position of SAAPs within Sri Lanka with Site1 and Site2. Molecular surface representation of *Pv*DBL with all polymorphic residue positions found in Sri Lankan isolates (golden rod) is shown in addition to Site1 (pink) and Site2 (purple) residues. Geographically exclusive polymorphic residues are labelled.

**Figure 6.**
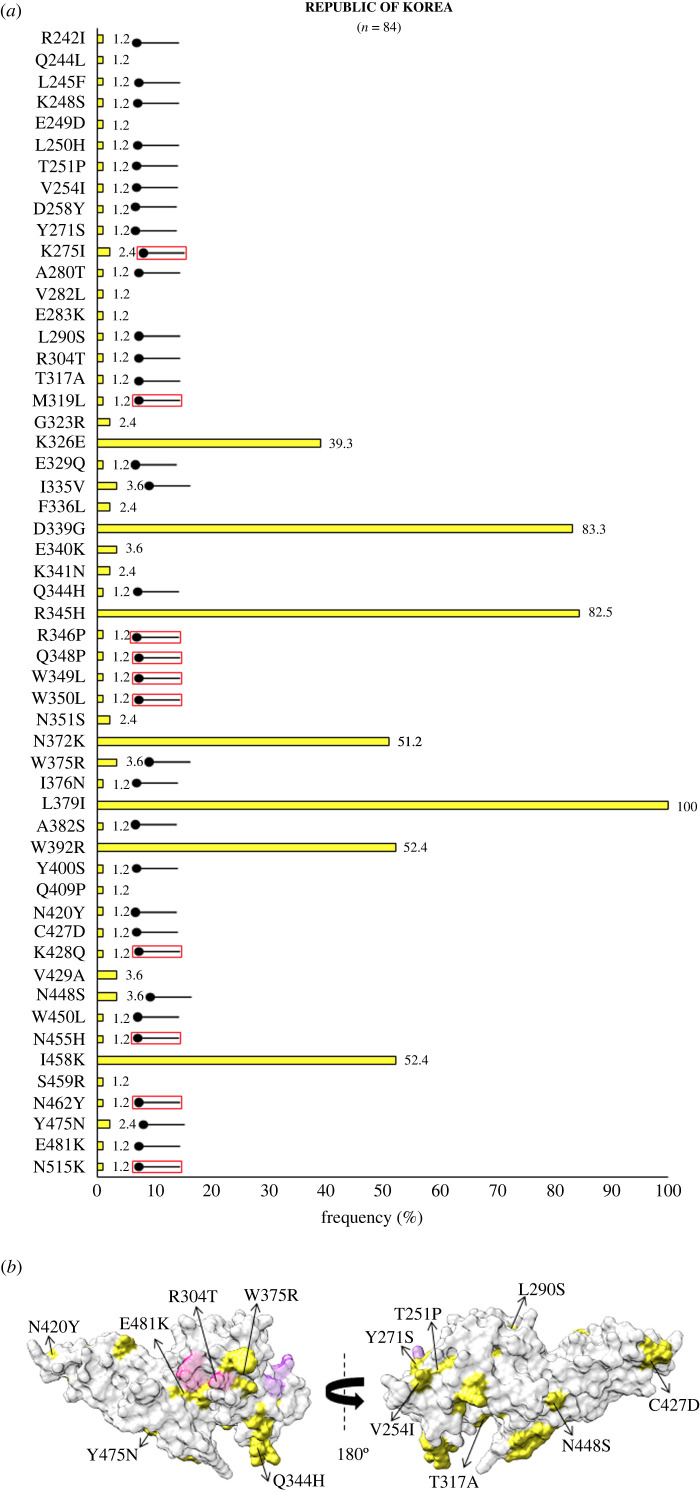
(*a*) Graph showing *Pv*DBL SAAP profile within Republic of Korea (RoK). Bar graph showing frequencies (yellow) of 54 polymorphic residues of *Pv*DBL observed in 84 sequences from RoK. Thirty-five geographically exclusive SAAPs observed in Korean isolates are marked with a horizontal balloon symbol. Of those 35, 25 are exclusive polymorphic residues while 10 are exclusive SAAP forms of multivariant residues (K275I, M319L, R346P, Q348P, W349L, W350L, K428Q, N455H, N462Y, N515K) and are marked with the help of a horizontal balloon outlined with red. (*b*) Relative structural position SAAPs within Republic of Korea with Site1 and Site2. Molecular surface representation of *Pv*DBL with all polymorphic residue positions found in Korean isolates (yellow) is shown in addition to Site1 (pink) and Site2 (purple) residues. Geographically exclusive polymorphic residues are labelled.

**Figure 7.**
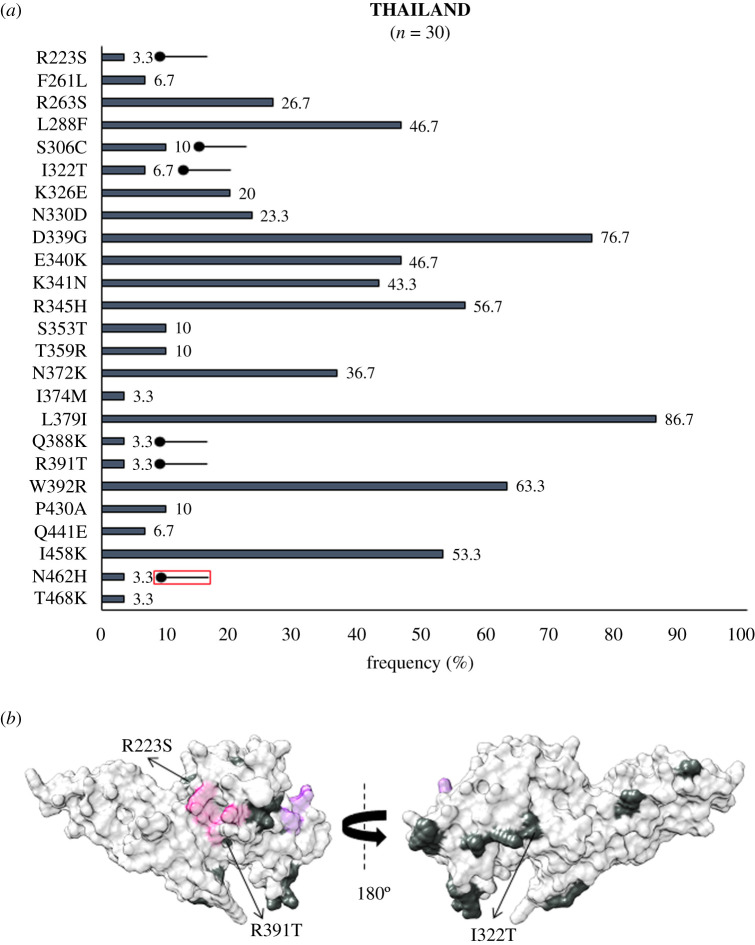
(*a*) Graph showing *Pv*DBL SAAP profile within Thailand. Bar graph showing frequencies (steel blue) of 25 polymorphic residues of PvDBL observed in 30 sequences from Thailand. Six geographically exclusive SAAPs observed in Thai isolates are marked with a horizontal balloon symbol. Of those six, five are exclusive polymorphic residues while one is an exclusive SAAP form of a multivariant residue (N462H) and is marked with the help of a horizontal balloon outlined with red. (*b*) Relative structural position of SAAPs within Thailand with Site1 and Site2. Molecular surface representation of *Pv*DBL with all polymorphic residue positions found in Thai isolates (steel blue) is shown in addition to Site1 (pink) and Site2 (purple) residues. Geographically exclusive polymorphic residues are labelled.

**Figure 8.**
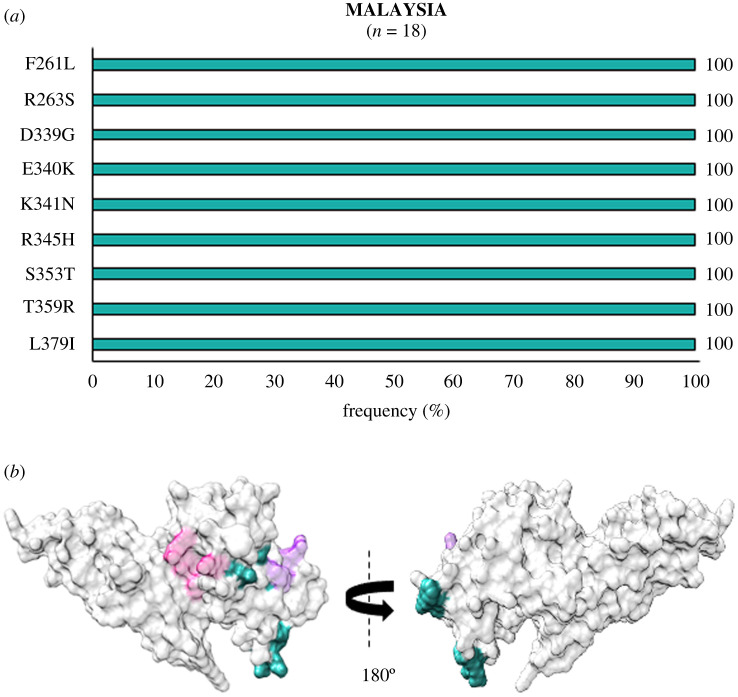
(*a*) Graph showing *Pv*DBL SAAP profile within Malaysia. Bar graph showing frequencies (dark cyan) of 9 polymorphic residues of *Pv*DBL observed in 18 sequences from Malaysia. No geographically exclusive SAAPs were found. (*b*) Relative structural position of SAAPs within Malaysia with Site1 and Site2. Molecular surface representation of *Pv*DBL with all polymorphic residue positions found in Malaysian isolates (dark cyan) is shown in addition to Site1 (pink) and Site2 (purple) residues.

In Southeast Asia within Myanmar, bordering India, 32 residue positions were observed to be polymorphic. The SAAPs K215E, I265L, F299S, K410I, K428R, C432G, R445K, D483G, K496T, A500V, A512T, N515I and V523G were geographically exclusive to Myanmar [39,67]. Of these residue positions, 410, 428 and 515 were found to be multivariant globally but these particular forms were found to be exclusive to Myanmar (figure 4*a*). The SAAP K410N was found in Iran, while K428Q and N515K manifest as singletons in isolates from the Republic of Korea (RoK). Among these, only F299S was found within 5 Å radius of Site1 (figure 4*b*). In Myanmar, the variants N372K, W392R and I458K were found to occur with frequencies of 66%, 70% and 55%, respectively. The trio of N372K-L379I-W392R occurred in 66% (146 of 221) isolates while 40% (88 of 221) isolates had the trio along with I458K. In Sri Lanka, which was declared malaria free in 2016, a total of 21 polymorphic residues were found from which three were geographically exclusive—N255Y, T444A and A463R/P (a trimorphic mutation) (figure 5*a*). The SAAP A463R/P maps to subdomain 3 away from Site 1 and 2 (figure 5*b*) [38]. A total of 36% (36 of 100) isolates exhibited the aforementioned trio. Moreover, 10% of isolates had the trio along with I458K. A total of 54 amino acid positions tend to be polymorphic within samples from the RoK. The geographically exclusive SAAPs found in RoK comprised R242I, L245F, K248S, L250H, T251P, V254I, D258Y, Y271S, K275I, A280T, L290S, R304T, T317A, M319L, E329Q, I335V, Q344H, R346P, Q348P, W349L, W350L, W375R, I376N, A382S, Y400S, N420Y, C427D, K428Q, N448S, W450L, N455H, N462Y, Y475N, E481K and N515K [40,64,71]. Of these 35 residue positions, the SAAPs at 10 positions—275, 319, 346, 348, 349, 350, 428, 455, 462 and 515—were multivariant globally but these particular forms were exclusive to isolates from RoK (figure 6*a*). R304T and E481K fall within 5 Å radius of Site1 whereas I335 V localizes to subdomain 2, being more proximal to Site2 than Site1 (figure 6*b*). The SAAP L379I was observed in all isolates from RoK. The quadruplet of N372K-L379I-W392R-I458K was found to occur in 50% (42 of 84) isolates from RoK. *Pv*DBL SAAP analyses from Thailand (SEA) revealed 25 residue positions to be polymorphic and several geographical unique SAAPs: R223S, S306C, I322T, Q388K and R391T. The position 462 was found to be multivariant globally but its N462H form was geographically exclusive to Thailand, whereas N462Y was found in isolates from RoK (figure 7*a*). Interestingly, S306C and R391T occurred within 5 Å radius of Site1 (figure 7*b*) [65]. Data from Malaysia was also found to be interesting wherein nine SAAPs—F261L, R263S, D339G, E340K, K341N, R345H, S353T, T359R, L379I (a unique haplotype)—were observed in all 18 protein sequences (figure 8*a*). F261L and R263S map to the disordered segment in between subdomains 1 and 2 while all other SAAPs localize to subdomain 2 (figure 8*b*).

**Figure 9.**
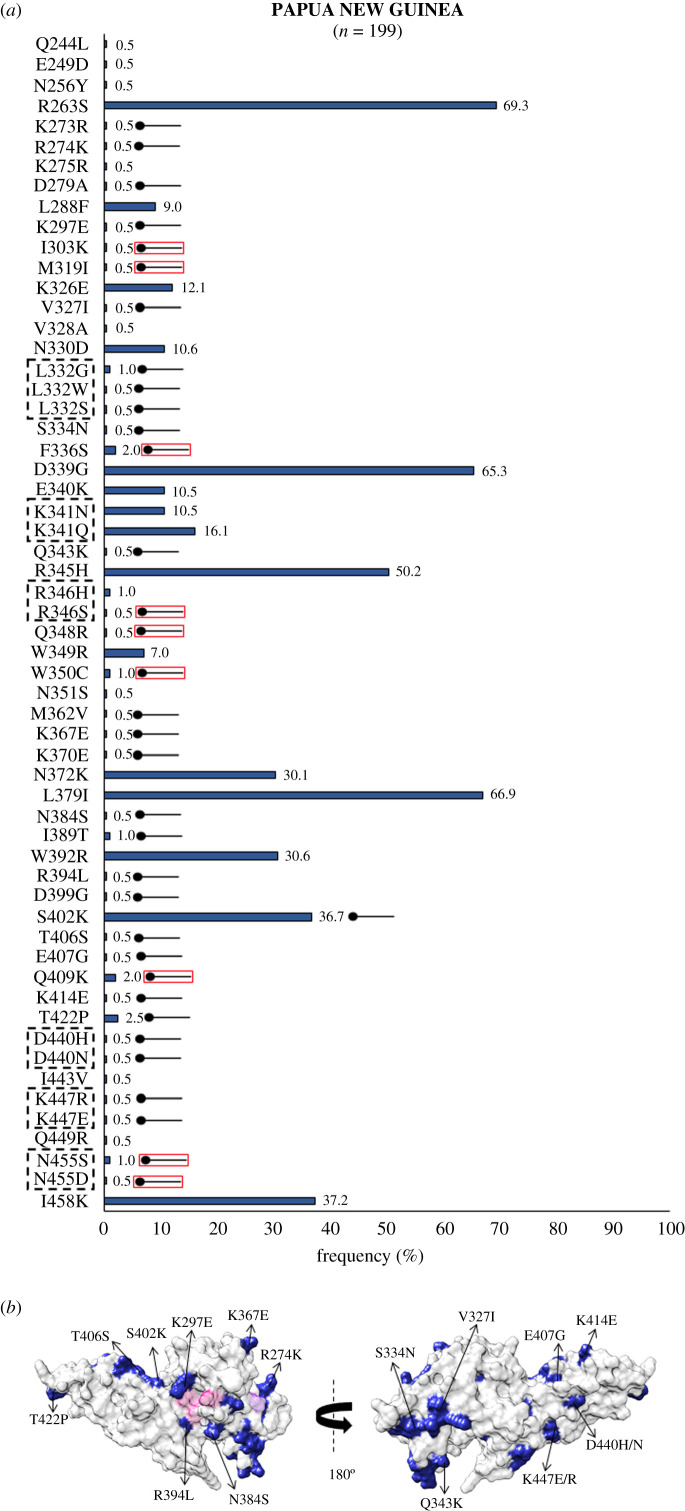
(*a*) Graph showing *Pv*DBL SAAP profile within Papua New Guinea. Bar graph showing frequencies (royal blue) of 58 SAAPs from 51 polymorphic residues of *Pv*DBL observed in 199 sequences from Papua New Guinea. Tri/tetra-morphic residues at positions 332, 341, 346, 440, 447 and 455 are outlined with a dashed rectangle. Thirty-five geographically exclusive SAAPs observed in sequences from Papua New Guinea are marked with a horizontal balloon symbol. Of those 35, 26 are exclusive polymorphic residues while 9 are exclusive SAAP forms of multivariant residues (I303K, M319I, F336S, R346S, Q348R, W350C, Q409K, N455S/D) and are marked with the help of a horizontal balloon outlined with red. (*b*) Relative structural position of SAAPs within Papua New Guinea with Site1 and Site2. Molecular surface representation of *Pv*DBL with all polymorphic residue positions found in Papua New Guinean isolates (royal blue) is shown in addition to Site1 (pink) and Site2 (purple) residues. Geographically exclusive polymorphic residues are labelled.

Within Oceania, *Pv*DBL sequence analysis from Papua New Guinea (PNG) revealed 51 polymorphic positions and several geographically exclusive SAAPs, as shown in figure 9, including K273R, R274K, D279A, K297E, V327I, L332G/W/S, S334N, Q343K, M362V, K367E, K370E, N384S, I389T, R394L, D399G, S402K, T406S, E407G, K414E, T422P, D440H/N and K447E/R [36,61,62]. Also, nine SAAPs occurred in multivariant forms globally while their particularly distinct forms were found to be unique to PNG—including I303K, M319I, F336, R346S, Q348R, W350C, Q409K and N455S/D. In relation to the aforementioned trio, approximately 30% (59 of 199) isolates exhibit this. Moreover, approximately 27% (53 of 199) isolates have I458 along with the trio. PNG therefore represents a unique SAAP profile of *Pv*DBL (figure 9*a*,*b*).

**Figure 10.**
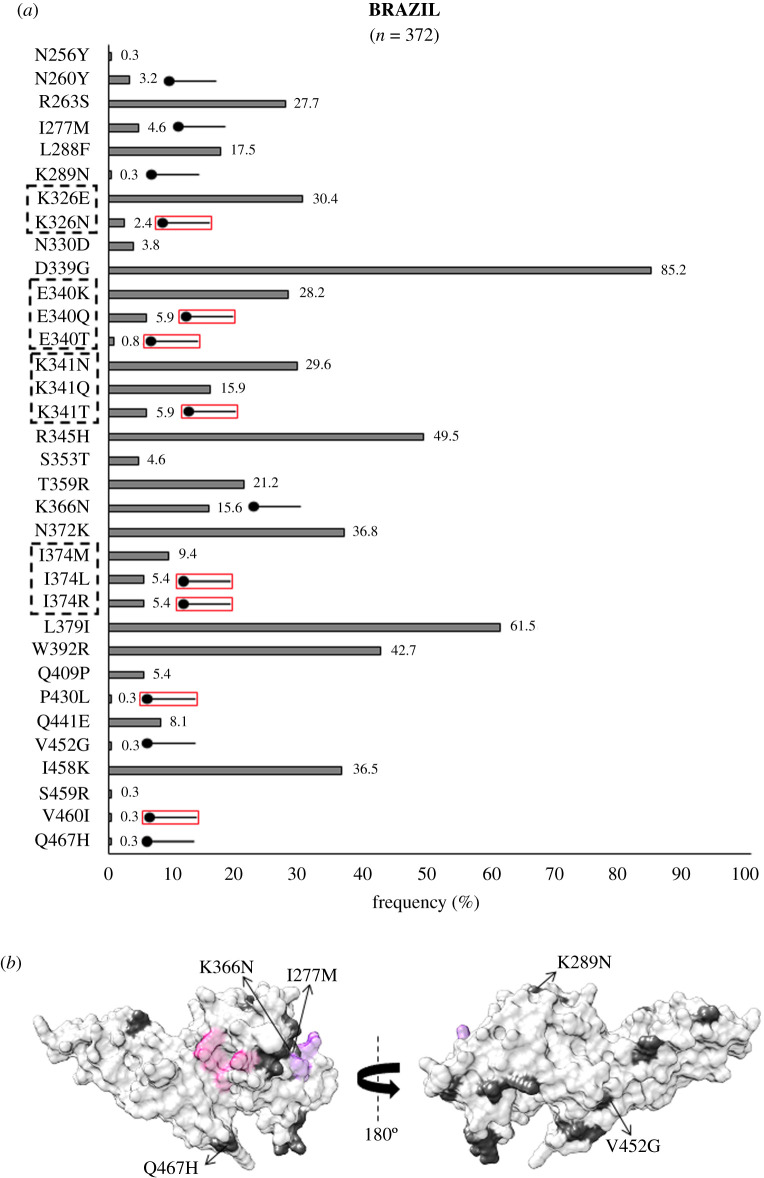
(*a*) Graph showing *Pv*DBL SAAP profile within Brazil. Bar graph showing frequencies (dim grey) of 34 SAAPs from 27 polymorphic residues of *Pv*DBL observed in 372 sequences from Brazil. Tri/tetra-morphic residues at positions 326, 340, 341 and 374 are outlined with a dashed rectangle. Fourteen geographically exclusive SAAPs observed in Brazil isolates are marked with a horizontal balloon symbol. Of those 14, 6 are exclusive polymorphic residues while 8 are exclusive SAAP forms of multivariant residues (K326N, E340Q, E340T, K341T, I374L, I374R, P430L, V460I) and are marked with the help of a horizontal balloon outlined with red. (b) Relative structural position of SAAPs within Brazil with Site1 and Site2. Molecular surface representation of *Pv*DBL with all polymorphic residue positions found in Brazilian isolates (dim grey) is shown in addition to Site1 (pink) and Site2 (purple) residues. Geographically exclusive polymorphic residues are labelled.

**Figure 11.**
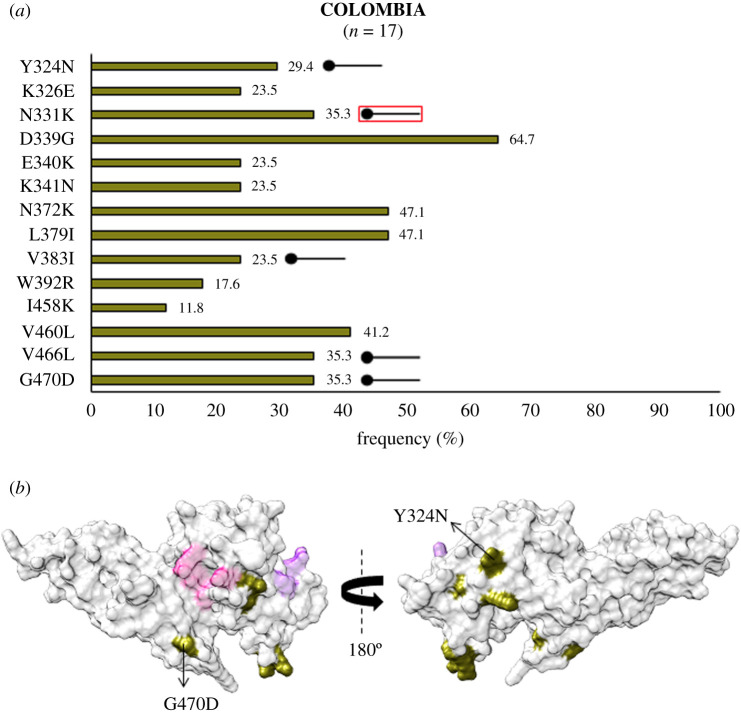
(*a*) Graph showing *Pv*DBL SAAP profile within Colombia. Bar graph showing frequencies (asparagus) of 14 SAAPs of *Pv*DBL observed in 17 sequences from Colombia. Five geographically exclusive SAAPs observed in Colombian isolates are marked with a horizontal balloon symbol. Of those five, four are exclusive polymorphic residues while one is exclusive SAAP form of a multivariant residue (N331K) and is marked with the help of a horizontal balloon outlined with red. (*b*) Relative structural position of SAAPs within Colombia with Site1 and Site2. Molecular surface representation of *Pv*DBL with all polymorphic residue positions found in Colombian isolates (asparagus) is shown in addition to Site1 (pink) and Site2 (purple) residues. Geographically exclusive polymorphic residues are labelled.

**Figure 12.**
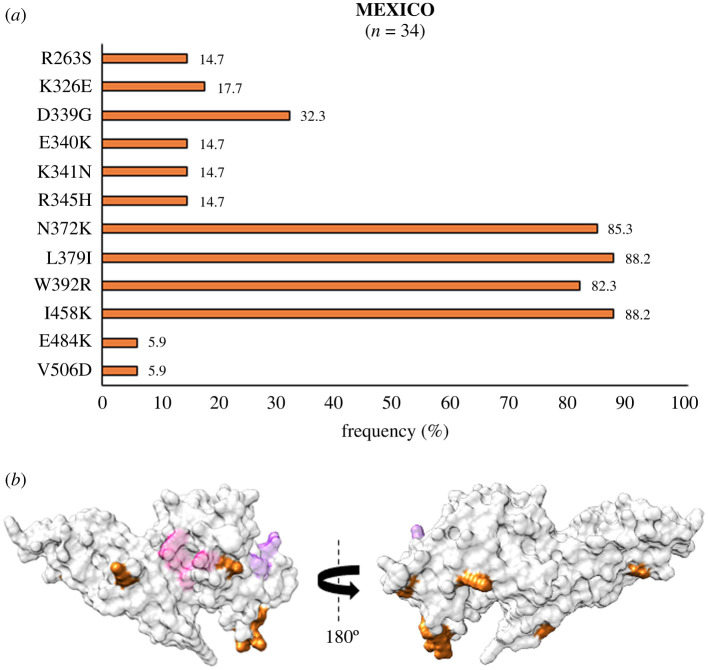
(*a*) Graph showing *Pv*DBL SAAP profile within Mexico. Bar graph showing frequencies (orange) of 12 polymorphic residues of *Pv*DBL observed in 34 sequences from Mexico. No geographically exclusive SAAPs were found. (*b*) Relative structural position of SAAPs within Mexico with Site1 and Site2. Molecular surface representation of *Pv*DBL with all polymorphic residue positions found in Mexican isolates (orange) is shown in addition to Site1 (pink) and Site2 (purple) residues.

In the Americas, analysis of *Pv*DBL sequences from Brazil revealed 27 polymorphic residues. Of these 27, amino acids at 326, 340, 341, 374, 430 and 460 were globally multivariant but one of their forms is unique to Brazil (as shown in figure 10*a*). SAAPs exclusive to Brazil include N260Y, I277M, K289N, K326N, E340Q/T, K341T, K366N, I374L/R, P430L, V452G, V460I and Q467H [37,70,75]. I374M/L/R was found to localize within 5 Å radius of Site1, while residue position 277 falls within 5 Å radius of Site2 (figure 10*b*). Some of the sequences also showed a leucine insertion between V429A and P430 as seen in isolates from India. The N372K-L379I-W392R trio was found in 37% (137 of 372) isolates while 17% (64 of 372) bear a fourth polymorphic residue, I458K, along with the trio. SAAP profiling from Colombia revealed Y324N localized to subdomain 2, V383I in the disordered region between subdomains 2 and 3 (occurred within 5 Å radius of Site1), and V466L with G470D in subdomain 3 were all geographically exclusive to this region (figure 11*a*, *b*) [69]. N331K was also exclusive to Colombia while N331I occurred as a singleton in isolates from India. Data from Mexico showed that there were no exclusive SAAPs [72]. We found no isolates from Mexico with the SAAP trio discussed previously while approximately 79% (27 of 34) isolates had the aforementioned SAAP quadruplet—N372 K-L379I-W392R-I458K (figure 12*a*,*b*).

**Figure 13.**
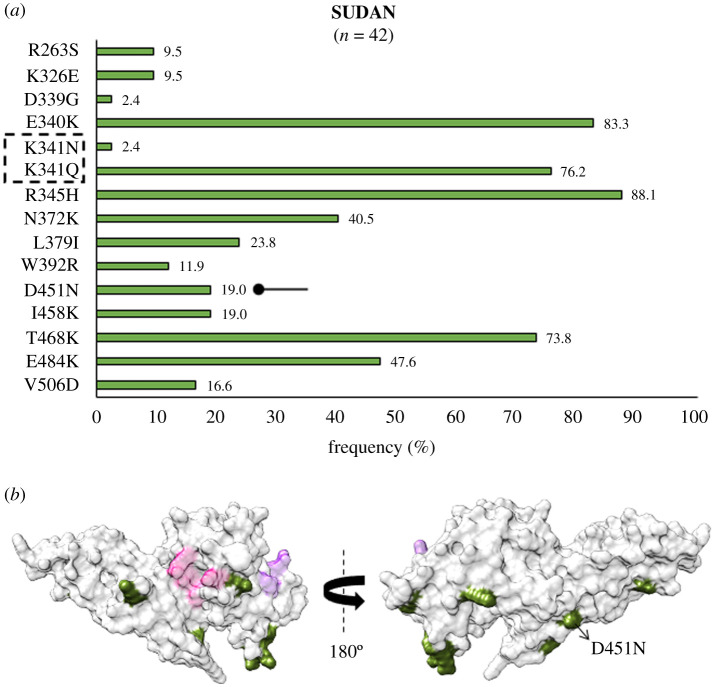
(*a*) Graph showing *Pv*DBL SAAP profile within Sudan. Bar graph showing frequencies (olive drab) of 15 SAAPs from 14 polymorphic residues of *Pv*DBL observed in 42 sequences from Sudan. One trimorphic residue at position 341 is outlined with a dashed rectangle. A geographically exclusive SAAP observed in Sudanese isolates is marked with a horizontal balloon symbol. (*b*) Relative structural position of SAAPs from within with Site1 and Site2. Molecular surface representation of *Pv*DBL with all polymorphic residue positions found in Sudanese isolates (olive) is shown in addition to Site1 (pink) and Site2 (purple) residues. Geographically exclusive polymorphic residues are labelled.

**Figure 14.**
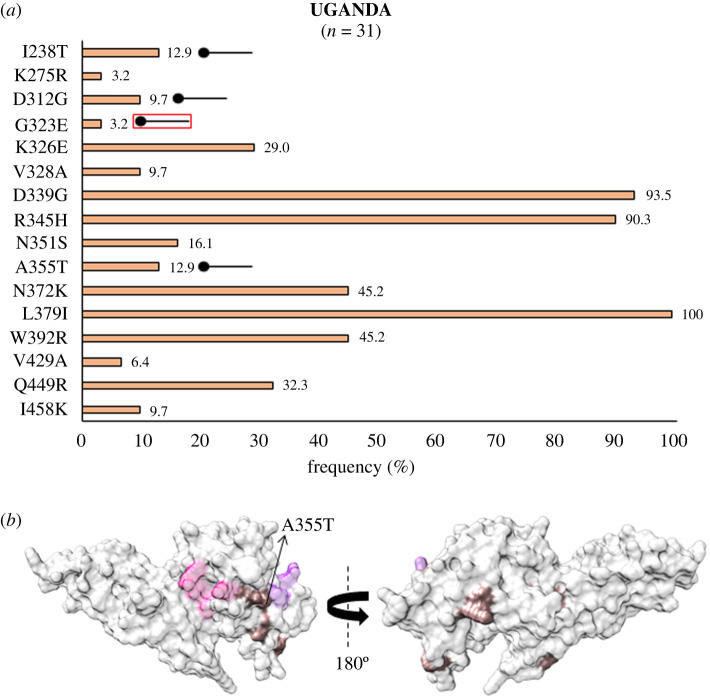
(*a*) Graph showing *Pv*DBL SAAP profile within Uganda. Bar graph showing frequencies (rosy brown) of 16 SAAPs of *Pv*DBL observed in 31 sequences from Uganda. Four geographically exclusive SAAPs observed in Ugandan isolates are marked with a horizontal balloon symbol. Of those four, three are exclusive polymorphic residues while one is an exclusive SAAP form of a multivariant residue (G323E) and is marked with the help of a horizontal balloon outlined with red. (*b*) Relative structural position of SAAPs within Uganda with Site1 and Site2. Molecular surface representation of *Pv*DBL with all polymorphic residue positions found in Ugandan isolates (rosy brown) is shown in addition to Site1 (pink) and Site2 (purple) residues. Geographically exclusive polymorphic residues are labelled.

From Africa, *Pv*DBL sequences from Sudan showed a similar profile of SAAPs as that of Mexico in comparison to other countries (besides other SAAPs, E484K and V506D were found to be polymorphic only within Sudan and Mexico) (figure 13*a* and *b*). D451N occurred as an exclusive SAAP found only in isolates from Sudan [74]. Intriguingly, up to approximately 76% (32 of 42) samples from Sudan had the SAAP quadruplet—N372K-L379I-W392R-I458K. We found 16 residues to be polymorphic in Uganda with I238T, D312G and A355T as geographically exclusive SAAPs (figure 14*a*). G323E was exclusive to Uganda in this form while its other polymorphic form G323R was found in isolates from RoK and India. Of these, only A355T falls within 5 Å radius of Site2 (figure 14*b*). L379I was found to occur in all the isolates from Uganda and approximately 45% (14 of 31) isolates displayed the trio, N372K-L379I-W392R, while approximately 32% (10 of 31) isolates have I458K as well.

**Figure 15.**
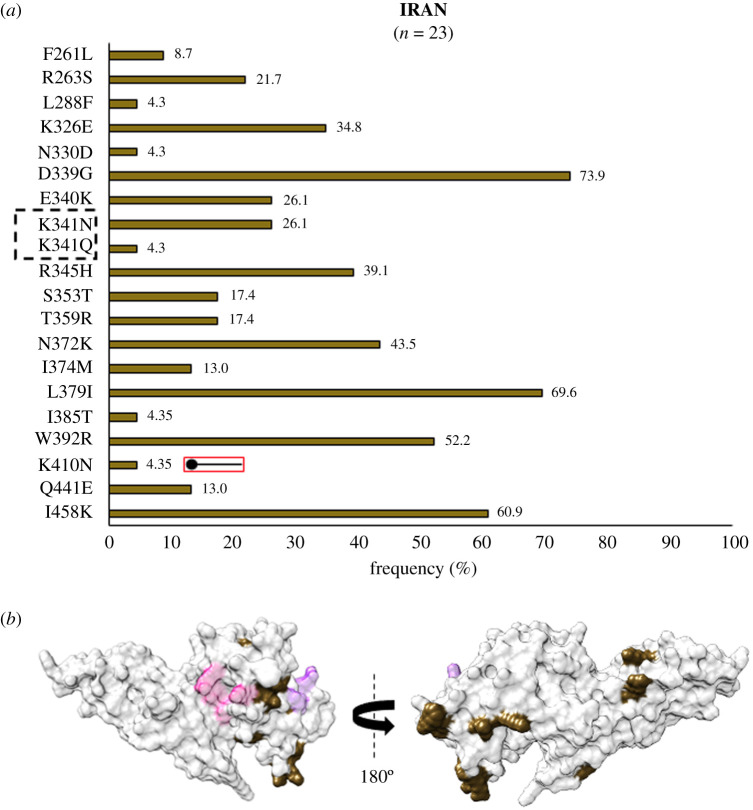
(*a*) Graph showing *Pv*DBL SAAP profile within Iran. Bar graph showing frequencies (brown) of 20 SAAPs from 19 polymorphic residues of *Pv*DBL observed in 23 sequences from Iran. One trimorphic residue at position 341 is outlined with a dashed rectangle. No geographically exclusive SAAPs are observed in Iranian isolates but an exclusive SAAP form of a multivariant residue (K410N) is marked with the help of a horizontal balloon outlined with red. (*b*) Relative structural position of SAAPs within Iran with Sites 1 and 2. Molecular surface representation of PvDBL with all polymorphic residue positions found in Iranian isolates (brown) is shown in addition to Site1 (pink) and Site2 (purple) residues.

Iran, an endemic area in the middle east, showed a unique SAAP profile with 19 polymorphic residues. Residue position 410 was multivariant—although its K410N form was found to be exclusive to Iran (figure 15*a*,*b*). A leucine insertion was also observed between V429 and P430 in subdomain 3, away from Site1 and Site2—as in India and Brazil [41,73,76,77].

**Figure 16.**
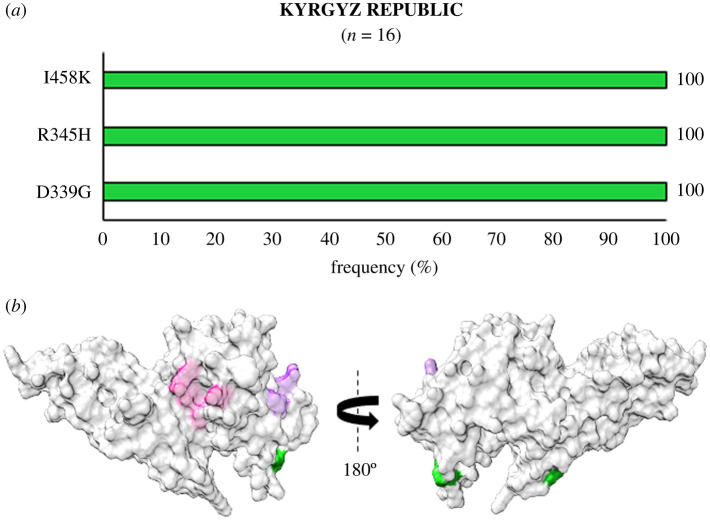
(*a*) Graph showing *Pv*DBL SAAP profile within Kyrgyz Republic. Bar graph showing frequencies (spring green) of 3 polymorphic residues of *Pv*DBL observed in 16 sequences from Kyrgyz Republic. No geographically exclusive SAAPs were found. (*b*) Relative structural position of SAAPs within Kyrgyz Republic with Sites 1 and 2. Molecular surface representation of *Pv*DBL with all polymorphic residue positions found in isolates from Kyrgyz Republic (spring green) is shown in addition to Site1 (pink) and Site2 (purple) residues.

Kyrgyz Republic from central Asia certified malaria free in 2016 by WHO, also displayed a very unique SAAP profile (figure 16*a*,*b*). Within the 16 sequenced samples, only three major SAAPs were found to occur—D339G, R345H and I458K—resulting in a unique haplotype which reveals much lower *Pv*DBL polymorphism in the Kyrgyz Republic as compared to other geographical regions [42].

### 3.3. Global SAAP profile and implications for vaccine development

**Figure 17.**
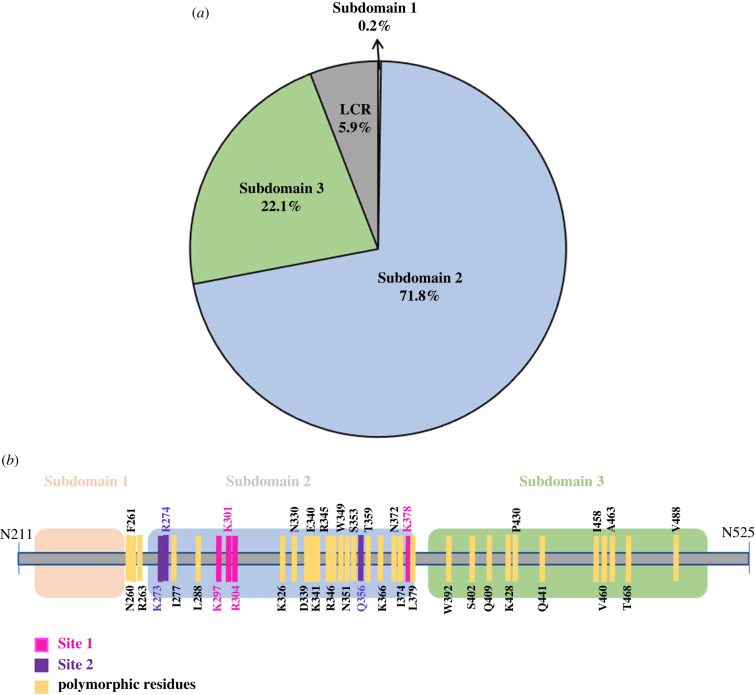
Distribution of SAAPs in *Pv*DBL subdomains and their schematic representation on domain diagram. (*a*) Pie chart showing distribution of instances of SAAPs in *Pv*DBL in terms of protein subdomains is shown. Subdomain 1 (salmon), Subdomain 2 (light blue), Subdomain 3 (light green) and LCR (grey) distributions have been labelled. (*b*) Schematic representation of *Pv*DBL domain architecture with Subdomain 1 (salmon), Subdomain 2 (light blue) and Subdomain 3 (light green) showing SAAPs that occur with frequencies greater than 0.5% globally (yellow), Site1 (pink) and Site2 (purple) residues.

**Figure 18.**
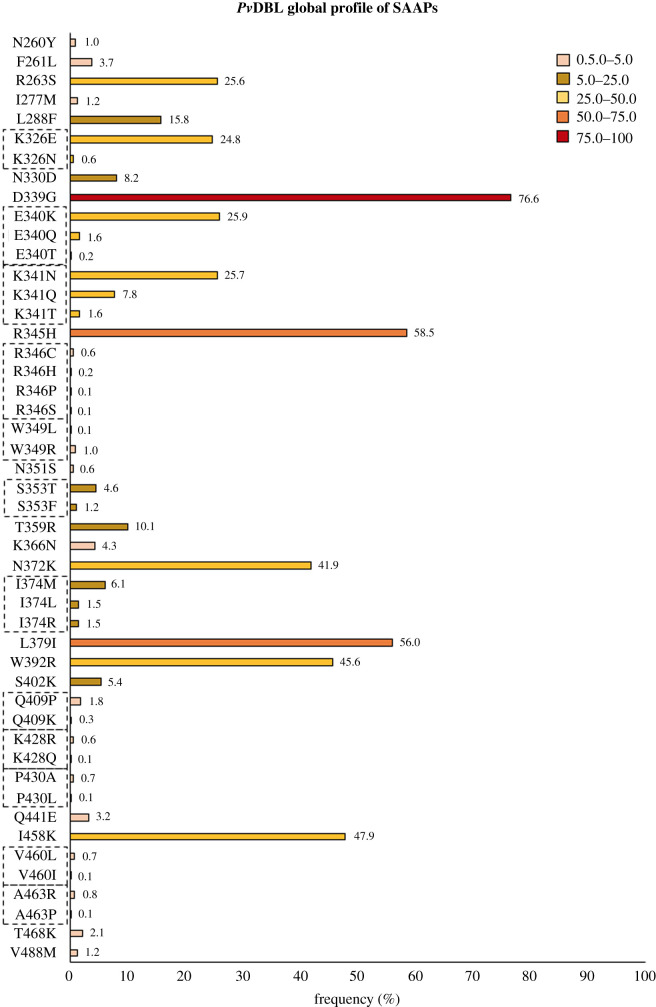
Global SAAP profile of *Pv*DBL. Graphical representation of SAAPs observed in *Pv*DBL with frequency greater than 0.5% within our global dataset of 1358 sequences. Tri-/tetra-/penta-morphic residues have been marked with a dashed rectangle around their residue labels. Histograms are colour coded according to frequencies: 1.0–5.0 (rose brown), 5.0–25.0 (golden rod), 25.0–50.0 (yellow), 50.0–75.0 (orange) and 75.0–100 (red). The frequencies of polymorphic residue SAAP forms are labelled on the top of corresponding histograms.

We observed that although the cysteine residues in *Pv*DBL were highly conserved, as much as half of all other constituent amino acids show polymorphism (i.e. 140 out of 315). Of these 140 polymorphic residues, approximately 40% were singletons i.e. occurred only once in a total set of 1358 sequences and were distributed randomly on the three-dimensional structure of *Pv*DBL. There were a total of 7298 instances of SAAPs observed in our dataset, out of which approximately 72% of instances of SAAPs occurred in subdomain 2 indicating its highly polymorphic nature when compared to the other two subdomains (0.2% and approximately 22% in subdomains 1 and 3, respectively, figure 17*a*). Some SAAPs (approx. 6%) which include two minor ones—N260Y and F261L—and one major—R263S—occurred outside the three subdomains in low complexity regions (LCR) (figure 17*a* and *b*). Thirty-one polymorphic residues were noted to occur with a frequency of at least 0.5% (figure 18). The subdomain-wise distribution of the 31 polymorphic positions on *Pv*DBL was approximately 53% (17 of 31) in subdomain 2, approximately 37% (11 of 31) in subdomain 3 and 10% (3 of 31) in the LCR. Of the 31, 16 occurred with a frequency of at least 5%. Two-thirds of the 31 residues were observed to be dimorphic while twelve residues at positions 326, 340, 341, 346, 349, 353, 374, 409, 428, 430, 460 and 463 show tri-, tetra-or penta-morphic changes (figure 18 and electronic supplementary material, table S1). Of these 31 polymorphic residues, three residues displayed conservative changes while 24 residues displayed non-conservative changes.

It was noted that of the multivariant residue positions 326, 340, 341, 374, 430 and 460 found in many geographical regions, their distinct forms K326N, E340Q/T, K341T, I374L/R, P430L and V460I were found exclusively within Brazil. Similarly, W349R was exclusively found in PNG but its other form W349L was unique to RoK. S353F was found to be exclusive to isolates from India. K428R was unique in isolates from Myanmar while its other form, K428Q, was found only in Korean isolates. The polymorphic residue A463R/P which showed a trimorphic change was uniquely observed in isolates from Sri Lanka. S402K occurred as a globally major SAAP while being found to be exclusive to PNG. I277M and K366N were globally minor SAAPs which occurred exclusively in isolates from Brazil.

**Figure 19.**
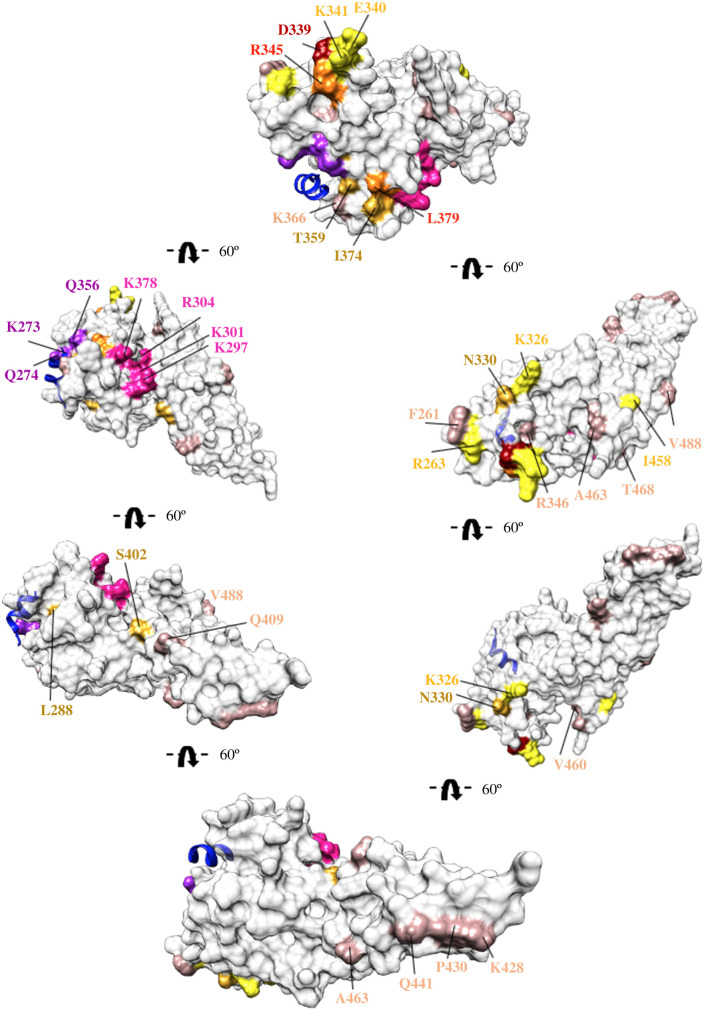
Molecular surface representation of *Pv*DBL showing relative position of global SAAPs in relation to Site1 and Site2. Surface representation of *Pv*DBL (grey) showing Site1 (pink) and Site2 (purple) residues. The structure is rotated 60° to cover the entire 360° view. Bound DARC peptide as per PDB ID: 4NUV is shown in blue. Amino acid residues that exhibit polymorphisms are coloured in accordance to Figure 18. Residues with frequencies 1–5% (rosy brown), 5–25% (golden rod), 25–50% (yellow), 50–75% (orange) and 75–100% (red) are labelled accordingly.

The locations of major and minor SAAPs do not overlap with the DARC engaging pockets called Site1 and Site2 (figure 19) [23,25]. A distinct clustering of four major SAAPs—D339G, E340K/Q/T, K341N/Q/T and R345H—at a conformational epitope—was found to be distal and opposite to Site1 and Site2 (figure 19), in agreement with the original hypothesis that immune evasion by the parasite is most likely facilitated by polymorphic clustering on regions away from DARC recognition residues [11]. The above-mentioned residues are part of an immunodominant epitope which may be employed as a decoy by the parasite to evade host immune response. A *Pv*DBL construct lacking the charged and polar residues in this epitope, known as DEK-null, reduces the immunogenicity of this immune-evading epitope and has been hypothesized to be a valid vaccine candidate [78].

Two major polymorphisms—S353F/T in Subdomain 2 and W392R in Subdomain 3—were found to be buried deep within the protein core. S353T/F was noted to occur within 5 Å radius of Site2 while W392R is closer to Site1 than Site2 [25]. The effects of swapping a hydrophobic residue (W392R) to a basic one or that of a polar residue to a hydrophobic one (S353T/F) are likely to be drastic. How this may change the topology of *Pv*DBL remains unanswered.

**Figure 20.**
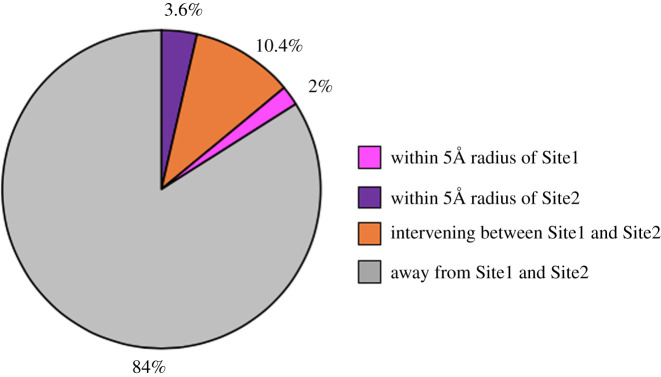
Distribution of SAAPs in vicinity of Site1 and Site2 of *Pv*DBL. Pie chart showing distribution of SAAPs according in the vicintiy of Site1 and Site2. Majority of SAAPs (84%) occur away from Site1 and Site2 (grey) while 10.4% map to the intervening space between Site1 and Site2 (orange). Only 2% occur within 5 Å radius of Site1 (pink) whereas 3.6% occur within 5 Å radius of Site2 (purple).

Our analyses reveal that only 16% instances of total SAAPs occur in the vicinity of Site1 and Site2, and the vast majority (84%) were spread throughout the surface of the *Pv*DBL molecule (figure 20). Among those in the vicinity of the two sites, 2% instances of total SAAPs fall within 5 Å radius of Site1 and 3.6% map within 5 Å radius of Site2 (table 3). Of the DbCRs, the SAAPs K273R and R274K in Site2 and K297E and R304T in Site1 are observed once each (1/1358 sequences). It is intriguing that the functionally conserved Site2 has more instances of SAAPs in its 5 Å radius than Site1 (3.6% versus 2%). Among the DaBRs, Y295, N296, L369 and I376 fall proximal to Site1 while K289, Y363, K366 and K367 are closer to Site2 while F299 and F373 fall in the space between Sites 1 and 2 (see electronic supplementary material, figure S1). Half of the DaBRs display polymorphisms albeit with varying frequencies—K289N (1/1358), F299S (5/1358), K366N (58/1358), K367E (1/1358) and I376N (1/1358). One major SAAP (L379I) maps to the intervening structural spaces between Site1 and Site2 and contributes 10.4% of total SAAP instances. It is noted that four SAAPs—I277M, W349R (both minor SAAPs), S353T and T359R (both major SAAPs)—occurred within 5 Å of Site2, whereas only one major SAAP—I374M—was found to occur within 5 Å radius of Site1 indicating higher physico-chemical conservation of the local environment of Site1 over Site2 and its importance as a key DARC-binding region, in agreement with previously published studies [11,14,18,22,23,25,27,28].

**Table 3.** Occurrence of SAAPs within the 5 Å radius of Site1 and Site2 of *Pv*DBL.

Site1		Site2	
*Pv*DBL residue	occurrence	*Pv*DBL residue	occurrence
Y295	NA	F267	NA
N296	NA	R268	NA
K297E	1	K269	NA
D298	NA	L270	NA
F299S	5	Y271S	1
C300	NA	L272	NA
K301	NA	K273R	1
D302	NA	R274 K	1
I303K/R	(1K + 2R) 3	K275R/I	(2R + 2I) 4
R304T	1	L276	NA
W305	NA	I277M	17
S306C	3	Y278	NA
L307	NA	D279A	1
G308	NA	W349R/L	(14R + 1L) 15
D309	NA	E352	NA
W358	NA	S353T/F	(62T + 16F) 78
I374M/L/R	(83M + 20L + 20R) 123	K354	NA
W375R	3	A355T	4
I376N	1	Q356	NA
C377	NA	I357	NA
K378	NA	W358	NA
N380	NA	T359R	137
V381	NA	A360	NA
A382S	1	M361	NA
V383I	4		
E386D	1		
R391T	1		
R394L	1		
R398	NA		
E481K	1		
total no. of instances within 5 Å of Site1	149	total no. of instances within 5 Å of Site2	259
total no. of instances	7298	total no. of instances	7298
percentage (%)	2.0%	percentage (%)	3.6%

Three major SAAPs—N372K, L379I (in Subdomain 2) and W392R (in Subdomain 3)—seem to be closely related as they occurred together in 43% of all the isolates analysed across different geographical regions. Moreover, 28% had the aforementioned trio in addition to I458K (in Subdomain 3). Among these, only L379 occurred in the intervening structural region between Site1 and Site2. N372 was found to be proximal to Site2, W392 was buried closer to Site1 in comparison to Site2, and I458 was distal to both Site1 and Site2 [25]. Previous studies have demonstrated the role of the SAAP trio N372K-W392R-I458K—particularly for residues W392 and I458 [43]. These are part of the dominant neutralizing epitopes that can change the antigenic character of DBP and alter the efficacy of immune inhibition [43].

### 3.4. Polymorphisms in epitopes of broadly neutralizing strain-transcending mAbs

**Figure 21.**
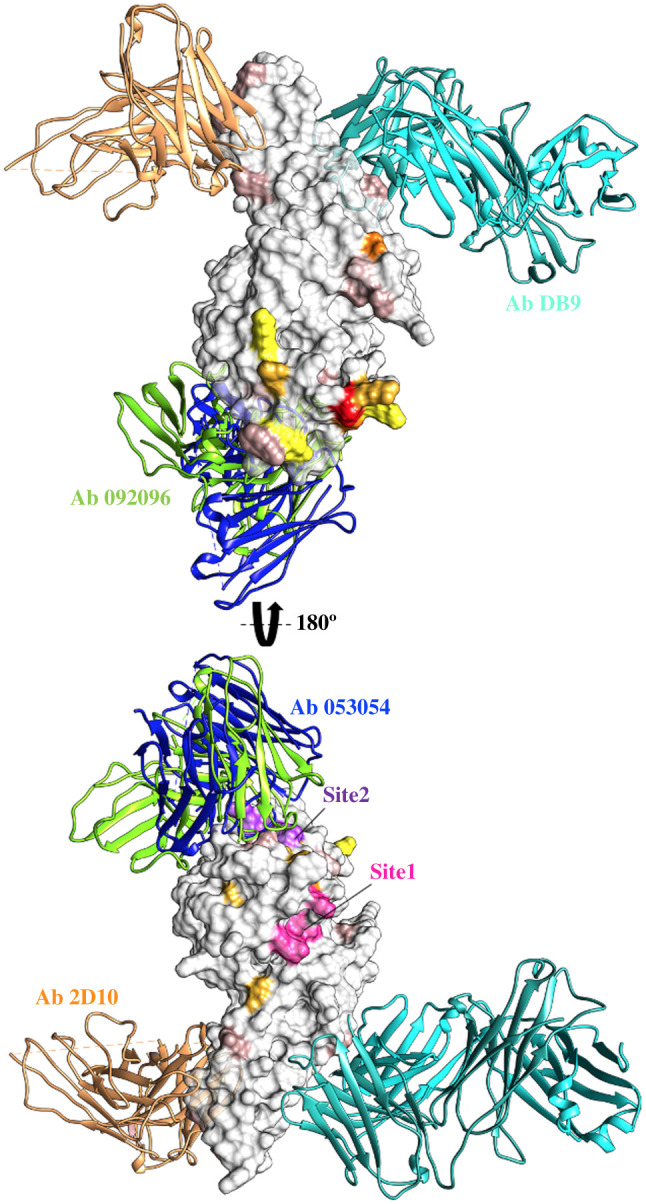
Molecular surface representation of *Pv*DBL showing relative positions of epitopes of 4 mAbs in relation to Site1, Site2 and global SAAPs. Surface representation of PvDBL (grey) bound to 4 mAbs purportedly having broadly neutralizing strain-transcending activity is shown. Antibodies from PDB IDs 5F3J (antibody 2D10, sandy brown), 6OAO (antibody 092096, chartreuse), 6OAN (antibody 053054, dark slate blue) and 6R2S (antibody DB9, light sea green) have been displayed to show their binding sites in relation to globally major SAAPs (colour coded in accordance with figure 18), Site1 (pink) and Site2 (purple) residues.

There are four *Pv*DBL–mAb complex structures submitted in the PDB, each purportedly with broadly neutralizing strain-transcending activity (PDB IDs: 5F3J, 6OAN, 6OAO, 6R2S) [79–81]. It is notable that in all the four complexes, the *Pv*DBL itself is monomeric and not dimeric [11,25]. Analyses of each of these was performed in detail to assess their interface binding regions and the location of DARC binding sites 1 and 2 in the context of the neutralizing mAb footprints (figure 21).

**Figure 22.**
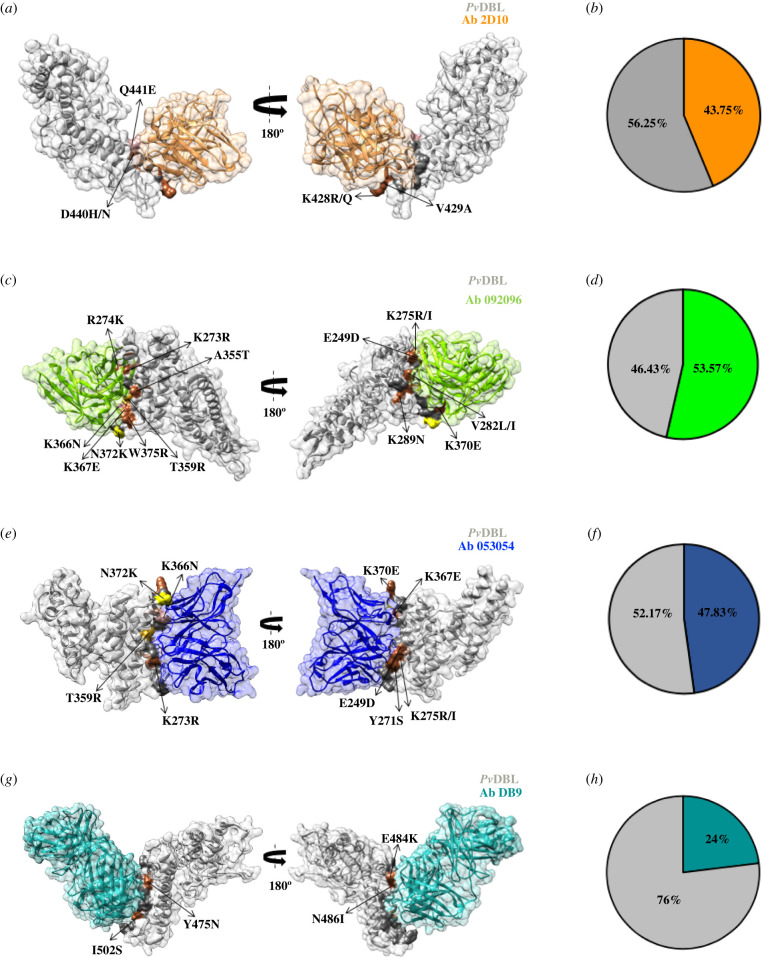
(*a*) Structural and graphical distribution of polymorphic residues within *Pv*DBL interface with mAb 2D10. Surface representation of *Pv*DBL (grey) in complex with a murine monoclonal antibody 2D10 (sandy brown) from PDB 5F3J is shown. Interface residues (dark grey), polymorphic interfacial residues (sienna) and global interfacial SAAPs (colour coded according to figure 18) are shown. Polymorphic residues around the interface are labelled. (*b*) Pie chart showing fraction of interface residues from 5F3J that are polymorphic (sandy brown). (*c*) Structural and graphical distribution of polymorphic residues within *Pv*DBL interface with mAb 092096. Surface representation of *Pv*DBL (grey) in complex with a human monoclonal antibody 092096 (chartreuse) from the PDB 6OAO. Interface residues (dark grey) and polymorphic residues (sienna) and global interfacial SAAPs (colour coded according to figure 18) are shown. Polymorphic residues around the interface are labelled. (*d*) Pie chart showing the fraction of interface residues from 6OAO that are polymorphic. (*e*) Structural and graphical distribution of polymorphic residues within *Pv*DBL interface with mAb 05304. Surface representation of *Pv*DBL (grey) in complex with a human monoclonal antibody 053054 (dark slate blue) as in the PDB 6OAN. Interface residues (dark grey) and polymorphic residues (sienna) and global interfacial SAAPs (colour coded according to figure 18) are shown. Polymorphic residues around the interface are labelled. (*f*) Pie chart showing the fraction of interface residues from 6OAN that are polymorphic. (*g*) Structural and graphical distribution of polymorphic residues within *Pv*DBL interface with mAb DB9. Surface representation of *Pv*DBL (grey) in complex with a human monoclonal antibody DB9 (light sea green) as in the PDB 6R2S. Interface residues (dark grey) and polymorphic residues (sienna) are shown. Polymorphic residues around the interface are labelled. (*h*) Pie chart showing the fraction of interface residues from 6R2S that are polymorphic.

The oldest of these *Pv*DBL–mAb structures is with a potent inhibitory murine monoclonal antibody 2D10 (PDB: 5F3J) whose epitope lies within subdomain 3 and was suggested to be conserved [81,82]. However, we show here that approximately 44% of the interfacing residues in *Pv*DBL exhibited a tendency to be polymorphic (figure 22*b*). Indeed, some of these SAAPs interact directly and could alter the binding efficiency of the antibody (table 4). Q441E, K428R/Q and P430A/L are globally minor SAAPs found at the interface of this epitope (figure 22*a*). Interestingly, an insertion of leucine was observed between V429 and P430 in isolates from Brazil, India and Iran that might potentially change the epitope conformation drastically thus affecting antibody binding efficacy.

**Table 4.** Frequency of SAAPs within epitope of murine mAb 2D10 (PDB: 5F3J).

*Pv*DBL residue	occurrence	frequency (%)^a^	type of change (C/NC)^b^	interaction between DBL and mAb	geographical distribution
E413		0			
K414E	1	0.07	NC	hydrogen bond	Papua New Guinea
D416		0			
G417		0			
K425		0		hydrogen bond	Myanmar
K428R/Q	10 (9R + Q)	0.74	C/NC		Myanmar (9R), Korea (1Q)
V429A	5	0.37	C		RoK (3), Uganda (2)
P430A/L	10	0.74	NC/NC	hydrogen bond	Brazil, Thailand, Myanmar
P431		0			
C432G	3	0.22	NC		Myanmar
Q433		0		hydrogen bond	
N434		0		hydrogen bond	
K437		0		salt bridge	
S438		0		hydrogen bond	
D440H/N	2	0.15	NC		Papua New Guinea
Q441E	44	3.24			global SAAP

**^a^**Frequency (%) = occurrence ÷ total no. of sequences (1358) × 100. **^b^**C = conservative change; NC = non-conservative change.

Three more recent *Pv*DBL–mAb structures are now available that dissect the structural basis of *Pv* neutralization with naturally acquired [79,83] or vaccine-induced human antibodies against *Pv*DBP [80]. Two of the three structures were with mAbs 092096 (PDB: 6OAO) and 053054 (PDB: 6OAN) that bind to the same face of *Pv*DBL and their epitopes show substantial overlap (figure 21) [79]. These epitopes lie in subdomain 2 and partly engage with *Pv*DBL Site2 residues, thereby neutralizing *Pv* by targeting the proposed dimer interface of *Pv*DBL [79,83,84]. Closer inspection of the *Pv*DBL–mAb interface revealed that approximately 54% and 48% of these interface residues (from antibody 092096 and 053054, respectively) tend to be polymorphic (figure 22*c*,*d*,*e*,*f*). There was high variability in the topology of recognized epitopes. T359R and N372K are two major SAAPs at the interfaces between *Pv*DBL and mAbs 053054 and 092096 (figure 22*c*,*e*). Urusova *et al.* suggest that these two mutations individually or in combination with other SAAPs like R263S, L288F or I374M do not affect the binding of antibodies to *Pv*DBL [79]. However, there are other SAAPs at the interface to be considered that include E249D, Y271S, K273R, R274K, K275R/I, I277M, V282L/I, A355T, K366N, K367E, K370E and W375R (tables 5 and 6). Among these, I277M and K366N are ‘minor’ SAAPs and are geographically exclusive to Brazil. Although other SAAPs occurred with low frequencies globally, they do exist in *Pv* isolates from multiple geographical regions. Most of the polymorphisms observed were non-conservative and therefore may alter the antigenic profiles of *Pv*DBL. Additionally, it has been previously reported that when N372K occurs with W392R and I458K, it compromises the efficiency of immune inhibition [43].

**Table 5.** Frequency of SAAPs within epitope of naturally acquired human mAb 092096 (PDB: 6OAO). See table 4 for notes on frequency and type of change.

*Pv*DBL residue	occurrence	frequency (%)	type of change (C/NC)	interaction between DBL and mAb	geographical distribution
Y219		0			
E249D	2	0.15	C		Papua New Guinea, RoK
L270		0			
Y271S	1	0.07	NC		RoK
K273R	1	0.07	C		Papua New Guinea
R274K	1	0.07	C	hydrogen bond	Papua New Guinea
K275R/I	4 (2R + 2I)	0.29	C/NC	hydrogen bond	RoK (2I), PNG (1R), Uganda (1R)
I277M	17	1.25	NC		Brazil
Y278		0		hydrogen bond	
A281		0			
V282L/I	4 (3L + I)	0.29	C/C		India, RoK
D285		0			
K289N	1	0.07	NC	hydrogen bond	Brazil
A355T	4	0.29	NC		Uganda
Q356		0		hydrogen bond	
T359R	137	10.09	NC	hydrogen bond	global SAAP
A360		0			
Y363		0		hydrogen bond	
S364		0			
K366N	58	4.27	NC	hydrogen bond and salt bridge	Brazil
K367E	1	0.07	NC	salt bridge	Papua New Guinea
R368		0		hydrogen bond	
L369		0			
K370E	1	0.07	NC		Papua New Guinea
G371		0			
N372K	569	41.90	NC		global SAAP
F373		0			
W375R	3	0.22	NC	hydrogen bond	RoK

**Table 6.** Frequency of SAAPS within epitope of naturally acquired human mAb 053054 (PDB: 6OAN). See table 4 for notes on frequency and type of change.

*Pv*DBL residue	occurrence	frequency (%)	type of change (C/NC)	interaction between DBL and mAb	geographical distribution
E249D	2	0.15	C		Papua New Guinea, RoK
D264		0			
T266		0			
F267		0			
L270		0			
Y271S	1	0.07	NC		RoK
K273R	1	0.07	C		Papua New Guinea
R274K	1	0.07	C	hydrogen bond	Papua New Guinea
K275R/I	4 (2R + 2I)	0.29		hydrogen bond	Papua New Guinea, Uganda
I277M	17	1.25	NC		Brazil
Y278		0		hydrogen bond	
A281		0			
Q356		0		hydrogen bond	
T359R	137	10.09	NC	hydrogen bond	major SAAP
A360		0			
Y363		0		hydrogen bond	
K366N	58	4.27	NC	hydrogen bond and salt bridge	Brazil
K367E	1	0.07	NC	salt bridge	Papua New Guinea
R368		0		hydrogen bond	
L369		0			
K370E	1	0.07	NC		Papua New Guinea
G371		0			
N372K	569	41.90	NC		major SAAP

Analysis of the fourth *Pv*DBL–mAb structure (PDB: 6R2S) with mAb DB9 revealed a similar trend of partial conservation of the recognized epitope despite its presence within subdomain 3 [80]. Approximately 24% of the interface residues here were also found to be polymorphic, with one globally minor SAAP V488M (figure 22*g*,*h*; table 7).

**Table 7.** Frequency of SAAPs within epitope of vaccine-induced human mAb DB9 (PDB: 6R2S). See table 4 for notes on frequency and type of change.

*Pv*DBL residue	occurrence	frequency (%)	type of change (C/NC)	interaction between DBL and mAb	geographical distribution
**at interface of heavy chain**					
L404		0			
V408		0			
K412		0		hydrogen bond	
D416		0			
K418		0			
Y421		0			
K424		0			
E484K	6	0.44	NC	hydrogen bond and salt bridge	Sudan, Mexico
N486I	1	0.07	NC	hydrogen bond	India
A489		0		hydrogen bond	
E493		0		hydrogen bond	
D498		0			
G499		0		hydrogen bond	
A500V	2	0.15	NC	hydrogen bond	Myanmar
I502S	1	0.07	NC		India
E503		0		hydrogen bond	
L504		0			
**at interface of light chain**					
Y475N	2	0.15	NC		RoK
D476		0		hydrogen bond	
K479		0			
E487		0		hydrogen bond	
N495		0			
**at interface of both heavy and light chain**					
V488M	17	1.25	NC		
N492		0		hydrogen bond	
R497		0		hydrogen bond	

Since residues that are critical for binding of antibodies have a tendency to be polymorphic, assessing their contribution to antibody-based neutralization becomes essential using a much wider landscape of SAAPs from field isolates. Besides, given the extremely low and non-proportional coverage of polymorphic space in terms of representation of sequences from *Pv* field isolates, conclusions about any new SAAPs being present in *Pv* endemic regions cannot be drawn. These analyses therefore suggest that deployment of any potential *Pv*DBL vaccine is unrealistic unless a greater extent of sequence variability has been mapped in *Pv* affected regions of the world.

## 4. Discussion

This study is the first to investigate the SAAP profile from India which contributes approximately half of the global *Pv* burden [56]. A limiting factor of this analysis is the assumption that sequence isolates from GenBank are from samples with mono-infection of *Pv* and no mixed samples were involved. A comparison of the most common amino acid changes in *Pv*DBL among presently studied *Pv* populations revealed that isolates from India showed a different SAAP profiling as compared to isolates from other geographical regions. The non-synonymous mutations Q409P, K428Q/R, P430A, V460L, A463R/P and T468K were found in other Southeast Asian regions but not in India. Moreover, in all other geographic regions, serine at position 353 changes into threonine, conservatively, whereas in India it changes non-conservatively into phenylalanine. We observed a leucine insertion between V429 and P430 in isolates from India, Brazil and Iran as well suggesting that these mutational events in geographically distant regions might be originating independently.

For *Pv*DBL, there seem to be currently two vaccine development strategies. The first entails developing a protein vaccine that encompasses most or all of the sequence variants present in endemic regions [66,78,81]. As highlighted, this will certainly not be an efficacious or cost-effective process given the sparse sampling of the *Pv*DBL sequence diversity studied so far. Intriguingly, Afghanistan, Ethiopia, Indonesia and Pakistan are also *Pv* endemic regions but no *Pv*DBL SAAP data are available for these regions in public databases. Altogether, this paucity of coverage will make it difficult to assess the feasibility of any *Pv*DBL-based vaccine. Periodic surveillance of *Pv*DBL polymorphisms across the whole *Pv* endemic space is essential. These analyses also call attention to the issue of implicit bias with regard to the country/region for which the DBL-based vaccine development effort is focused at. The sequence space that has so far addressed polymorphisms in *Pv*DBL is very limited, but despite this, varying profiles of SAAPs are evident in different *Pv* endemic regions of the world. The evident diversity just within Asia is indicative of the immense difficulty in designing a single subunit vaccine capable of covering the full spectrum of variations in the isolates of *Pv* in the context of their DBL sequences.

The second strategy purports the use of the antigen with conserved B-cell epitopes that can overcome strain-specific immunity [78,82,83]. Thus, broadly neutralizing antibodies raised against a globally conserved epitope would be the basic requirement for the rational design of a strain-transcending DBL-based vaccine [82]. From the four *Pv*DBL–mAb complex structures, it is evident that two of the four purported neutralizing mAbs do not bind near the supposed dimer interface. Further, although it has been suggested that the above mAbs bind to conformational epitopes that are broadly neutralizing and hence the possible target of strain-transcending global protection, we show via an in-depth global SAAP analysis, that this may not be so. The polymorphisms observed at amino acid positions 372, 379, 392 and 458 might be due to immune pressure that aids the parasite to evade host immunity. This pressure generates new *Pv*DBL variants that are still functional but adept at escaping inhibitory antibodies. It is noteworthy that host immunity evasion due to SAAPs in *Pv*DBL are predicted to hamper binding efficacy of neutralizing antibodies that recognize conformational epitopes due to a change in topological features; however, these are not confirmed outcomes.

This work therefore suggests that no single *Pv*DBL sequence may be used as a platform for vaccine development as the inherent variability in *Pv*DBL sequences will render such vaccines inefficacious. A global real-time database needs to be built from *Pv* afflicted regions to assess the current state and spread of polymorphic *Pv* strains, and their respective DBL sequences to discern presently conserved epitopes that may be targeted by neutralizing antibodies. Moreover, the presence of *Pv* in DARC negative populations along with the recent finding that gene amplification could be an additional immune evasion mechanism used by *Pv* emphasizes the importance of a multicomponent vaccine strategy that in addition to eliciting inhibitory antibodies may also reduce the ability of the parasite to escape immunological control [85]. In sum, this study suggests the need of a vast expansion and analysis of the *Pv*DBL sequence database, region- and country-wise, in order to assess the real feasibility of *Pv*DBL as a vaccine against *Pv* malaria.

## Supplementary Material

List of all SAAPs of PvDBL

## Supplementary Material

Molecular surface representation of PvDBL with Site1, Site2 and DaBRs

## Data accessibility

DNA sequences: GenBank accession numbers MT502426–MT502498. The relevant sequences will be publicly available at the NCBI portal after May 2021.
