# Supplementary material for: Global distribution of single amino acid polymorphisms in Plasmodium vivax Duffy-binding-like domain and implications for vaccine development efforts
Source: Open Biol. 2020 Sep 30;10(9):200180. doi: 10.1098/rsob.200180 (PMC7536081; doi:10.1098/rsob.200180)
Supplement: List of all SAAPs of PvDBL [file rsob200180supp1.docx]

**Supplementary Table T1**

**List of all SAAPs of *Pv*DBL**

| **S. No.** | **PvDBL Residue change** | **Occurrence** | **Conservative(C) or Non-Conservative Change (NC)** | **Frequency* (%)** |
| --- | --- | --- | --- | --- |
| 1. | K215E | 1 | NC | 0.07 |
| 2. | R223S | 1 | NC | 0.07 |
| 3. | D229G | 1 | NC | 0.07 |
| 4. | V236G | 1 | C | 0.07 |
| 5. | I238T | 4 | NC | 0.29 |
| 6. | D240E | 1 | C | 0.07 |
| 7. | R242I | 1 | NC | 0.07 |
| 8. | Q244L/H | 3 (2L + 1H) | NC/NC | 0.22 |
| 9. | L245F | 1 | NC | 0.07 |
| 10. | E249D | 2 | C | 0.15 |
| 11. | L250H | 1 | NC | 0.07 |
| 12. | T251P | 1 | NC | 0.07 |
| 13. | V252L | 1 | C | 0.07 |
| 14. | V254I | 1 | C | 0.07 |
| 15. | N255Y | 3 | NC | 0.22 |
| 16. | N256Y | 2 | NC | 0.15 |
| 17. | D258Y | 1 | NC | 0.07 |
| 18. | T259P | 1 | NC | 0.07 |
| 19. | N260Y | 14 | NC | 1.03 |
| 20. | F261L | 51 | NC | 3.75 |
| 21. | R263S | 348 | NC | 25.62 |
| 22. | I265L | 2 | C | 0.15 |
| 23. | Y271S | 1 | NC | 0.07 |
| 24. | K273R | 1 | C | 0.07 |
| 25. | R274K | 1 | C | 0.07 |
| 26. | K275R/I | 4 (2R +2I) | C/NC | 0.29 |
| 27. | I277M | 17 | NC | 1.25 |
| 28. | D279A | 1 | NC | 0.07 |
| 29. | A280T | 1 | NC | 0.07 |
| 30. | V282L/I | 4 (3L + I) | C/C | 0.29 |
| 31. | E283K | 2 | NC | 0.15 |
| 32. | L288F | 215 | NC | 15.83 |
| 33. | K289N | 1 | NC | 0.07 |
| 34. | L290S | 1 | NC | 0.07 |
| 35. | R294K | 1 | C | 0.07 |
| 36. | K297E | 1 | NC | 0.07 |
| 37. | F299S | 5 | NC | 0.37 |
| 38. | I303K/R | 3 (1K + 2R) | NC/NC | 0.22 |
| 39. | R304T | 1 | NC | 0.07 |
| 40. | S306C | 3 | C | 0.22 |
| 41. | D312G | 3 | NC | 0.22 |
| 42. | T317A | 1 | NC | 0.07 |
| 43. | M319L/I | 2 (L + I) | NC/NC | 0.15 |
| 44. | I322T | 2 | NC | 0.15 |
| 45. | G323R/E | 4 (3R + E) | NC/NC | 0.29 |
| 46. | Y324N | 5 | NC | 0.37 |
| 47. | K326E/N | 346 (337E + 9N) | NC/NC | 25.48 |
| 48. | V327I | 1 | C | 0.07 |
| 49. | V328A | 4 | C | 0.29 |
| 50. | E329Q | 1 | C | 0.07 |
| 51. | N330D | 111 | C | 8.17 |
| 52. | N331K/I | 7 (6K + I) | NC/NC | 0.51 |
| 53. | L332G/W/S | 4 (2G + W + S) | C/NC/NC | 0.29 |
| 54. | S334N | 1 | NC | 0.07 |
| 55. | I335V | 3 | C | 0.22 |
| 56. | F336L/S | 7 (3L + 4S) | NC/NC | 0.51 |
| 57. | D339G | 1040 | NC | 76.58 |
| 58. | E340K/Q/T | 377 (352K + 22Q + 3T) | NC/C/NC | 27.76 |
| 59. | K341N/Q/T | 477 (349N + 106Q + 22T) | NC/NC/NC | 35.12 |
| 60. | Q343K | 1 | NC | 0.07 |
| 61. | Q344H | 1 | NC | 0.07 |
| 62. | R345H | 794 | C | 58.47 |
| 63. | R346H/S/C/P | 14 (3H + 9C + S + P) | C/NC/NC/NC | 1.03 |
| 64. | Q348R/P | 2 (P + R) | NC/NC | 0.15 |
| 65. | W349L/R | 15 (L + 14R) | NC/NC | 1.10 |
| 66. | W350L/C | 3 (L + 2C) | NC/NC | 0.22 |
| 67. | N351S | 8 | NC | 0.59 |
| 68. | S353T/F | 78 (62T + 16F) | C/NC | 5.74 |
| 69. | A355T | 4 | NC | 0.29 |
| 70. | T359R | 137 | NC | 10.09 |
| 71. | M362V | 1 | NC | 0.07 |
| 72. | K366N | 58 | NC | 4.27 |
| 73. | K367E | 1 | NC | 0.07 |
| 74. | K370E | 1 | NC | 0.07 |
| 75. | N372K | 569 | NC | 41.89 |
| 76. | I374M/L/R | 123 (83M + 20L + 20R) | NC/C/NC | 9.06 |
| 77. | W375R | 3 | NC | 0.22 |
| 78. | I376N | 1 | NC | 0.07 |
| 79. | L379I | 761 | C | 56.04 |
| 80. | A382S | 1 | NC | 0.07 |
| 81. | V383I | 4 | C | 0.29 |
| 82. | N384S | 1 | NC | 0.07 |
| 83. | I385T | 4 | NC | 0.29 |
| 84. | E386D | 1 | C | 0.07 |
| 85. | Q388K | 1 | NC | 0.07 |
| 86. | I389T | 2 | NC | 0.15 |
| 87. | R391T | 1 | NC | 0.07 |
| 88. | W392R | 619 | NC | 45.58 |
| 89. | I393F | 1 | NC | 0.07 |
| 90. | R394L | 1 | NC | 0.07 |
| 91. | D399G | 1 | NC | 0.07 |
| 92. | Y400S | 1 | NC | 0.07 |
| 93. | S402K | 73 | NC | 5.37 |
| 94. | T406S | 1 | C | 0.07 |
| 95. | E407G | 1 | NC | 0.07 |
| 96. | Q409P/K | 29 (25P + 4K) | NC/NC | 2.13 |
| 97. | K410I/N | 4 (3I + N) | NC/NC | 0.29 |
| 98. | K414E | 1 | NC | 0.07 |
| 99. | N420Y | 1 | NC | 0.07 |
| 100. | T422P | 5 | NC | 0.37 |
| 101. | C427D | 1 | NC | 0.07 |
| 102. | K428R/Q | 10 (9R + Q) | C/NC | 0.70 |
| 103. | V429A | 5 | C | 0.37 |
| 104. | P430A/L | 10 | NC/NC | 0.74 |
| 105. | C432G | 3 | NC | 0.22 |
| 106. | D440H/N | 2 (H + N) | NC/C | 0.15 |
| 107. | Q441E | 44 | C | 3.24 |
| 108. | I443V | 3 | C | 0.22 |
| 109. | T444A | 3 | NC | 0.22 |
| 110. | R445K | 3 | C | 0.22 |
| 111. | K447E/R | 2 (E + R) | NC/C | 0.15 |
| 112. | N448S | 3 | NC | 0.22 |
| 113. | Q449R | 4 | NC | 0.29 |
| 114. | W450L | 1 | NC | 0.07 |
| 115. | D451N | 1 | C | 0.07 |
| 116. | V452G | 1 | C | 0.07 |
| 117. | N455H/S/D | 3 (H + S + D) | NC/NC/C | 0.22 |
| 118. | I458K | 650 | NC | 47.86 |
| 119. | S459R | 5 | NC | 0.37 |
| 120. | V460L/I | 11(10L+I) | C/C | 0.81 |
| 121. | N462Y/H | 2 (Y + H) | NC/NC | 0.15 |
| 122. | A463R/P | 13 (11R + 2P) | NC/NC | 0.96 |
| 123. | V466L | 6 | C | 0.44 |
| 124. | Q467H | 2 | NC | 0.15 |
| 125. | T468K | 29 | NC | 2.13 |
| 126. | G470D | 6 | NC | 0.44 |
| 127. | Y475N | 2 | NC | 0.15 |
| 128. | E481K | 1 | NC | 0.07 |
| 129. | D483G | 3 | NC | 0.22 |
| 130. | E484K | 6 | NC | 0.44 |
| 131. | N486I | 1 | NC | 0.07 |
| 132. | V488M | 17 | NC | 1.25 |
| 133. | F490L | 1 | NC | 0.07 |
| 134. | K496T | 3 | NC | 0.22 |
| 135. | A500V | 2 | C | 0.15 |
| 136. | I502S | 1 | NC | 0.07 |
| 137. | V506D | 6 | NC | 0.44 |
| 138. | A512T | 1 | NC | 0.07 |
| 139. | N515I/K | 2 (I + K) | NC/NC | 0.15 |
| 140. | V523G | 1 | C | 0.07 |
|  | **Total Occurrences** | **7,298** |  |  |

***Frequency (%) = Occurrence/Total No. of sequences (1358) *100**
