# Supplementary material for: Global distribution of single amino acid polymorphisms in Plasmodium vivax Duffy-binding-like domain and implications for vaccine development efforts
Source: Open Biol. 2020 Sep 30;10(9):200180. doi: 10.1098/rsob.200180 (PMC7536081; doi:10.1098/rsob.200180)
Supplement: Molecular surface representation of PvDBL with Site1, Site2 and DaBRs [file rsob200180supp2.docx]

**Supplementary Fig S1**


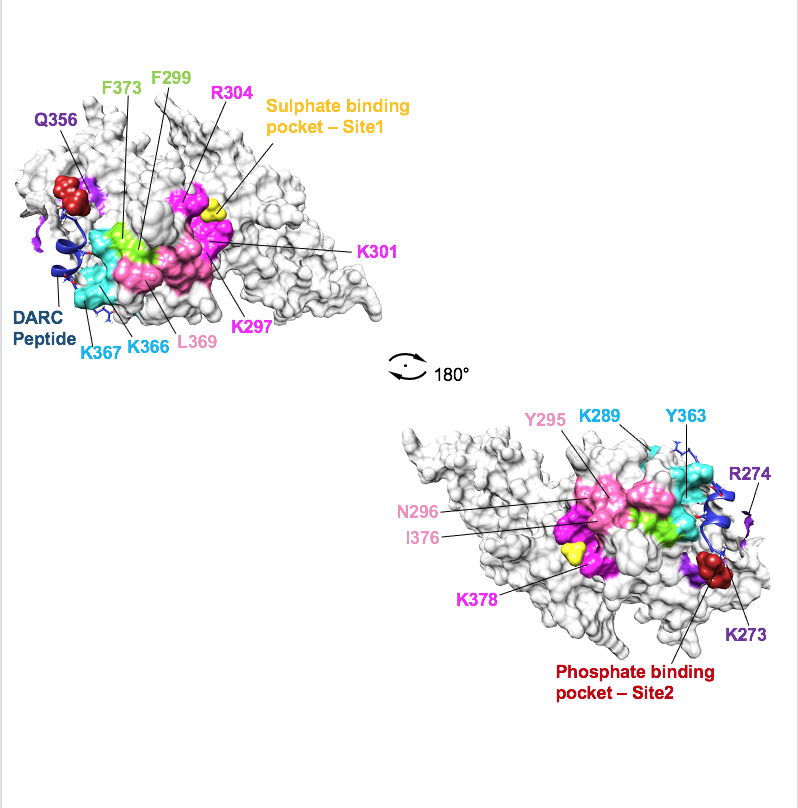


**Supplementary Fig S1**: Molecular surface representation of *Pv*DBL (light grey) and Site1 (Magenta) and Site2 (purple) are shown. The yellow colour depicts sulphate site for DARC- Tyr41 and in firebrick is the phosphate site with DARC-Tyr30. The hot pink colour denotes the non-polar and hydrophobic residues that are DARC-associated binding residues (DaBR) near Site1. In chartreuse, are the unique DaBR hydrophobic residues that likely maintain DARC in position for DARC-Tyr30 and DARC-Tyr41 to interact. In cyan are residues that are DaBR near Site2.
